# Interventions to support young carers/supporters of people living with dementia: a mixed methods systematic review

**DOI:** 10.1080/17482631.2026.2650367

**Published:** 2026-03-27

**Authors:** Kirstie Goodchild, Ellice Parkinson, Jane L Cross

**Affiliations:** a Faculty of Medicine and Health Sciences, Queens’ Building, University of East Anglia, Norwich, England, United Kingdom; b University of East Anglia and Health Innovation East, Norwich, England, United Kingdom; c University of East Anglia, Norwich, England, United Kingdom

**Keywords:** Dementia, children, dementia care-related interventions, young people, Alzheimer’s disease

## Abstract

**Purpose:**

Despite children being young carers for people living with dementia globally, and evidence suggesting they need more support, there is limited research evaluating best practice for dementia-care related interventions for children. The purpose of this work was therefore to comprehensively summarise the existing literature by synthesising studies appraising existing child-focused and dementia-care relevant interventions.

**Method:**

A mixed methods systematic review with a convergent integrated synthesis approach. Four databases were systematically searched from 1st January 2013 to 9th February 2024. Qualitative, quantitative, and mixed methods studies evaluating any intervention programme that aimed to improve children’s understanding and/or support for people living with dementia were included.

**Results:**

Seventeen studies, evaluating 15 different interventions (1,345 participants), were eligible for inclusion. Extracted data were inductively synthesised into 18 categories, forming six integrated findings relating to what makes interventions useful for helping children to understand and/or support people living with dementia.

**Conclusions:**

The findings can inform the development of interventions for children with dementia care responsibilities, and further robust research.

## Introduction

Dementia is a syndrome which impairs cognition and ‘everyday activity’ performance, and is usually chronic, progressive and more extensive than typical biological ageing (Alzheimer’s Association, 2021; WHO, 2022). People living with dementia increasingly require care at home from informal carers, which can include child-aged relatives (Bremer et al., 2017; Chirico et al., 2021; Connolly, 2020; Johansson et al., 2021; Tavares & Freitas, 2019). Young people may become involved in caregiving due to being motivated by their relationship with the person living with dementia, their will to avoid them moving into residential care, as well as adult informal caregivers potentially being unavailable (D’Amen et al., 2021). Children acting as young carers worldwide, mainly to parents and grandparents living with dementia, have consistently communicated the need for enhanced support within their caring roles, due to the impact of providing informal care on their lives, including to their psychological health, development, social opportunities and well-being (Becker, 2007; Chirico et al., 2021; Gelman & Rhames, 2018; Grundberg et al., 2021; Venters & Jones, 2021). A recently published systematic review highlights a particular scarcity of appropriate support for children of parents with young-onset dementia (occurring before age 65) (Cartwright et al., 2021). The estimated number of people living with dementia (worldwide was 50 million in 2018 and is predicted to increase to 152 million by 2050 (Alzheimer’s Disease International, 2018). As the frequency of children’s exposure to dementia is thus only likely to increase (Alzheimer’s Disease International, 2018; Carter et al., 2018), it is vital to address this lack of appropriate support for dementia young carers now to mitigate impacts on their health and well-being.

To date, systematic reviews of interventions aiming to support informal dementia carers have focused on adults, who make up the highest proportion of such caregivers (Boots et al., 2014; Egan and Pinto-Bruno, 2018; Klimova et al., 2019; Wolff et al., 2018). Consequently, the evidence base for interventions supporting children to care for people living with dementia is limited. Indeed, *iSupport,* the self-declared first virtual dementia intervention for 11-17-year-old informal carers, is yet to be widely implemented and evaluated (Masterson-Algar et al., 2023). The limited evidence and apparent lack of dementia care-related interventions for children means it is unclear what would be most important to include in a child-targeted intervention (Masterson-Algar et al., 2023). To address this question, this review asks, “*What makes interventions useful for helping children to understand and/or support people living with dementia*?”. Its overall aim was to comprehensively summarise the existing literature by synthesising studies appraising existing child-focused and dementia-care relevant interventions, to inform future intervention design and further research.

## Methods

### Design

This systematic review followed the *JBI methodology for Mixed Methods Systematic Reviews* (Silver & Francis, 2022) and adheres to the *Preferred Reporting Items for Systematic Reviews and Meta-Analyses (PRISMA) 2020 statement* (Page et al., 2021) (Supplementary Material: Appendix 1). The protocol was registered on the PROSPERO register of systematic reviews on 12^th^ March 2024 (registration number CRD42024516392), available at: https://www.crd.york.ac.uk/PROSPERO/display_record.php?RecordID=516392.

### Search strategy

**Figure 1.**
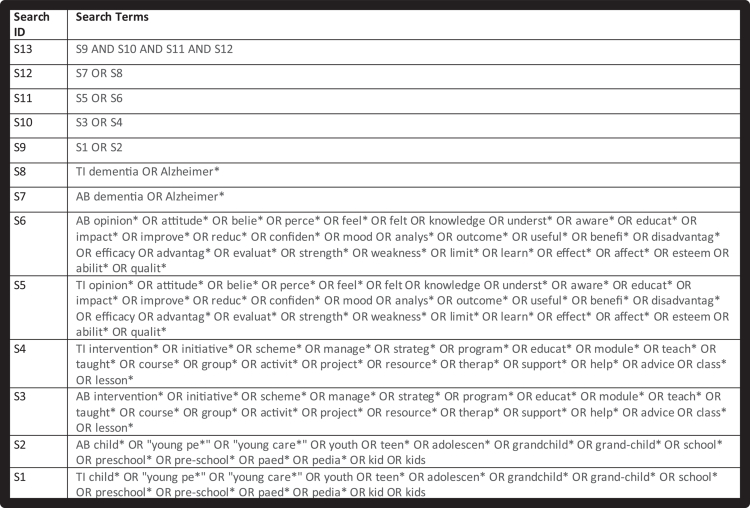
Search strategy summary.

MEDLINE (Ultimate), CINAHL Ultimate (EBSCO), APA PsycINFO and WebofScience were searched on 9^th^ February 2024. We developed the search strategy using an iterative process guided by (Bramer et al., 2018) and with support from a specialist university librarian. The full search strategy is summarised in Figure 1 and was tailored for each database (Supplementary Material (Appendix 2)). We restricted the search to English language and published from January 1^st^, 2013. We used reference and citation searching of the included studies’ reference lists to identify further eligible papers.

### Eligibility criteria

#### Population

Included studies reported outcomes of interest for children (<18 years old). We included studies which reported data for this population separate from other participant groups. Studies including students aged 18-19 years-year-olds were eligible for inclusion where > 50% of the sample were < 18 years. It was not possible to differentiate results from children with and without personal experience of dementia from the included studies.

#### Phenomena of interest

##### Exposure.

The review included studies evaluating dementia-care relevant interventions delivered in any setting. Interventions were eligible if they had content about supporting or understanding people living with any type of dementia of any severity. If a study explored interventions for various illnesses, the full-text was required to report dementia-specific outcomes which could be analysed independently of findings related to any other health conditions studied. If the study investigated different intervention programmes, the structures and outcomes for each needed to be reported separately.

##### Outcomes of interest.

Eligible studies presented qualitative and/or quantitative measures reporting on an intervention’s usefulness, defined as its ability to help children understand and/or support people living with dementia. Eligible outcomes measures reported usefulness using descriptive or numerical data retrieved from children directly. Outcome measures were only eligible for inclusion if collected after intervention delivery.

#### Types of studies

Any qualitative, quantitative or mixed methods primary research studies were included (Clarke, 2011; Silver & Francis, 2022). Eligible study designs included randomised control trials, quantitative non-randomised, quantitative descriptive, qualitative descriptive and grey literature studies. There were no restrictions on the place of publication. Sources unavailable in English and published before 2013 were excluded.

### Study selection

Search results were imported into Endnote 20 reference management software (The EndNote Team, 2013) for removal of duplicates. The first author screened all titles and abstracts, using the eligibility criteria, in EndNote. Any eligible titles and abstracts were then grouped in EndNote, and full texts searched and uploaded. The first author then screened all full texts against the eligibility criteria. The second author duplicated screening blindly for 20% of title and abstracts (589 papers), and then for 20% of full texts (nine papers), as per the pre-registered protocol. Seventeen studies were assessed eligible for inclusion. Any conflicts between consensus were discussed and resolved between the two reviewers.

### Assessment of methodological quality

The first author independently assessed included studies for their methodological quality using the *JBI Critical Appraisal Checklist for Qualitative Research* (JBI, 2017) for qualitative studies, and the *2018 Version of the Mixed Methods Appraisal Tool (MMAT)* (Hong et al., 2018) for quantitative and mixed methods studies. Quality appraisal questions were answered as *unclear* unless there was indisputable evidence for giving a positive or negative response. The second author duplicated critical appraisal for 20% of included studies (four papers). Due to the limited time frame of the review, study authors were not contacted to request missing or additional data for clarification. Instead, quality appraisal questions were answered as unclear unless there was indisputable evidence for giving a positive or negative response.

### Appraisal of study level of evidence

The Grading of Recommendations Assessment, Development and Evaluation (GRADE) (Ryan & Hill, 2016) and ConQual (Munn et al., 2014) systems were used to determine the level of evidence for included studies.

### Data extraction

The first author independently extracted relevant data from all sections of all included studies. Data were extracted into an Excel table with headings adapted from the JBI Mixed Methods Data Extract Form following a Convergent Integrated Approach (Silver & Francis, 2022). Extracted data included methods, sample sizes, participant ages, intervention details, relevant outcome measures and geographical and cultural context. Extracted quantitative outcome data included averages, percentages and inferential test results. These generally described participants’ scores or score changes in validated and bespoke questionnaires. Extracted qualitative data were descriptive results, themes and subthemes, and illustrative data supporting these findings, such as quotes. The second author duplicated blind data extraction in 20% of the included studies (four papers), to ensure key outcome data and study characteristics had been comprehensively extracted. In keeping with the eligibility criteria, only results measuring usefulness using data retrieved from children directly were extracted. Where relevant results were not statistically significant, these were still included for improved validity and comprehensiveness (Silver & Francis, 2022).

### Data transformation

In line with the chosen synthesis method, quantitative data were converted into *qualitized* data to help integrate quantitative and qualitative findings; this is deemed more accurate than converting qualitative into quantitative data (JBI, 2014; Silver & Francis, 2022). The *qualitizing* process involved transforming quantitative results into narrative interpretations or textual descriptions related to the research question (Supplementary Material: Appendix 3). Extracted qualitative data were interpreted and written as findings relevant to the research question (Supplementary Material: Appendix 3). To help visually display results from each study, each qualitative and qualitized finding was labelled with an alphanumeric code consisting of a letter representing the study it was extracted from, and a number representing each unique finding from the study (Gonella et al., 2022) (Supplementary Material: Appendix 3).

Each qualitative finding received a credibility rating by assessing the congruence between the finding and the supporting data (Silver & Francis, 2022). Possible rankings were *unsupported* (data do not support findings), *credible* (finding is plausible based on the data but challengeable) and *unequivocal* (unchallengeable data support findings) (Silver & Francis, 2022). Consistent with the review’s synthesis approach, any unsupported qualitative findings were excluded from the final synthesis (Silver & Francis, 2022).

### Synthesis

The findings were synthesised using the *convergent integrated approach,* recommended for mixed methods systematic reviews (Lizarondo et al., 2020). This synthesis approach examines findings in detail to combine qualitized and qualitative data into categories based on similarity. Categories can include qualitative or qualitized data, or a mixture of both. Aggregating similar categories creates integrated findings, producing the review’s results.

All the review’s eligible qualitative and qualitized findings were exported into a Microsoft Word document. The review team familiarised themselves with the data and rearranged them into categories. They then reviewed the categories to ensure they were supported by component findings, mutually exclusive, and represented one category only. This process was repeated until no new categories were generated. The categories were named to represent their component findings. The same overall process was applied to group the categories into defined integrated findings revealing what makes interventions useful for helping children understand and/or support people living with dementia. The review team examined categories and integrated findings against the data and refined over multiple meetings to ensure fit and agreement amongst all reviewers.

### Appraisal of level of evidence for integrated findings

The level of evidence for each integrated finding was assigned using the ConQual approach (Munn et al., 2014) to help aggregate component finding evidence levels, supplemented with researcher insight based on study quality and level of evidence appraisals, and evidence quantity (Gonella et al., 2022; Munn et al., 2014).

## Results

### Study inclusion

**Figure 2.**
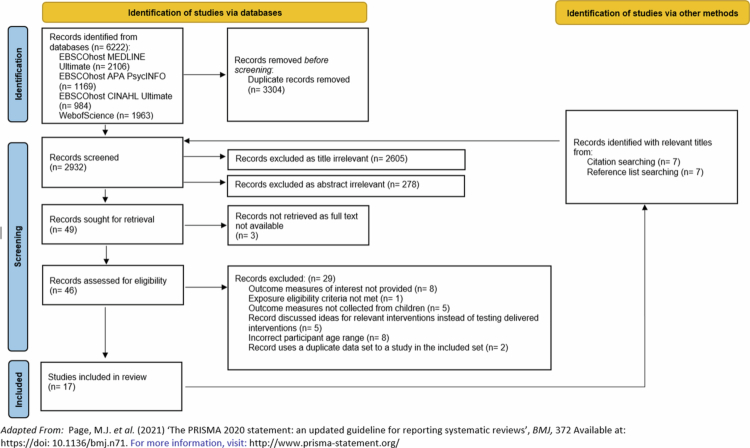
PRISMA flow diagram summarising the review’s screening process.

The searches retrieved 2932 unique records. After reviewing titles and abstracts, 49 were eligible for full-text screening, of which 17 were assessed as eligible for inclusion in the final review (Figure 2). Studies were excluded for multiple reasons including if relevant outcome measures were not provided or if a duplicate data set was used to that of another included study (Figure 2). The full list of excluded studies and the reasoning for exclusion is in the Supplementary Material (Appendix 4).

### Methodological quality

We assessed two studies as having moderate methodological quality, five as low, and ten as very low quality (Table I). The quality of all the studies was impacted by issues such as use of selective samples, lack of control of confounding variables and not all findings being unequivocal. Each study’s appraisal results and justifications are detailed in full in the Supplementary Material (Appendix 5).

**Table I.** Characteristics of the included studies.

Alpha Code: First Author (Year)	Publication	Peer-Reviewed?	Study Type	Methods	Number of Participants Relevant Outcome Measures were Collected From KEY: Total Number (Number of Females)^*^	Ages (years)	Evaluated Intervention’s Name^*^: Summary Including its Aims and Length.	Country, Geographical Setting and/or Cultural Context*	Relevant Outcome Measures	Level of Evidence^****^
**A: Baker et al. (2019)**	Dementia	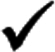	MM	Non-randomised waitlist-controlled pre-/post-intervention design. Three FGs.	**195** *Intervention group:* 135*(80)* *Waitlist group:* 60*(39)* *QL results:* 19	9−12	Kids4Dementia: teacher-led multimedia DEP to improve dementia literacy. 150 minutes delivered over 4 to 10 weeks.	**Australia.** Three independent Christian primary schools.	Kids Insight into Dementia Survey (KIDS) score changes. FG transcript content analysis results.	Low 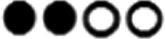
**B: Burns et al. (2021)**	Health Promotion Journal of Australia	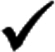	MM	Non-randomised simple within-subjects pre-/post-intervention design Artwork comparisons.	**109** *QL results:* 109 *QN results:* 74*(33)*	8−10	Project DARE: arts-based DEP to increase understanding about dementia. Three sessions delivered one day a week, over a three-week period. Each session lasted one hour.	**Australia.** Affluent primary school.	Modified KIDS score changes. Results of content analysis of 109 children’s pre- and post-intervention memory-related artworks.	Very Low 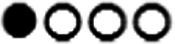
**C: Chow et al. (2018)**	Journal of Public Health	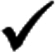	MM	Non-randomised simple within-subjects pre-/post-intervention design pilot study Surveys.	**4**	15−17	Dementia Awareness Programme: to improve dementia knowledge and appreciation, and provide intergenerational opportunities for students to volunteer with and enrich the lives of people with dementia. Weekly hour-long visits with a person living with dementia for a total of 15 weeks.	**Canada.** High school.	Dementia attitudes questionnaire*** responses. Facts on Aging Quiz score changes.	Very Low 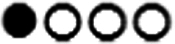
**D: Di Bona et al. (2019)**	Dementia	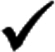	MM	Non-randomised simple within-subjects pre-/post-intervention design. One FG.	**41** *QN results:* 41 (excluded) *QL results:* 13	9−10	Adopt a Care Home: teacher-led DEP with intergenerational experience for some students to improve children’s dementia awareness and improve wellbeing and community participation for people living with dementia in care homes. Several care home visits of undisclosed length. One school term of regular dementia lessons.	**England.** Primary school. Care home. 69% white British.	Dementia Awareness Questionnaire score changes (excluded). FG transcript analysis results.	Very Low 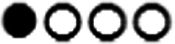
**E: Farina et al. (2020a)**	Public Health	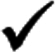	QL	Four FGs.	**30** *(15)*	11−16	Dementia Friends: dementia education and awareness initiative aimed at reducing dementia stigma. 45-60 minute long single session.	**England.** Two secondary schools. 89.7% White British participants.	FG transcript inductive thematic analysis results.	Moderate 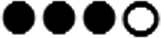
**F: Farina et al. (2020b)**	BMC Geriatrics	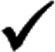	QN	Non-randomised pre-/post-intervention design with control group study.	**301***(172)* *Intervention group*: 198 *Control:* 102	12−16	Dementia Friends: dementia education and awareness initiative aimed at reducing dementia stigma. 45-60 minute long single session.	**England.** Secondary schools. 78.1% White British participants.	Brief Adolescent Attitudes towards Dementia Scale and KIDS score changes. Dementia career interest and intervention satisfaction survey*** scores.	Low 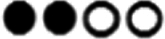
**G: Gibson et al. (2018)**	Dementia	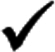	QL	Non-randomised simple within-subjects pre-/post-intervention design. Artwork- and writing-based QL outcomes.	**18**	5−17^**^	Adapted Dementia Friends: interactive dementia education and awareness initiative aimed at educating about how lives of people living with dementia can be improved. 150 minute long session with a break consisting of four 15 minute long interactive sessions.	**Scotland.** Various schools.	Results of thematic analysis of 18 children’s pre- and post-intervention dementia-related artwork and words/phrases they associate with dementia. Results of thematic analysis of 18 children’s words/phrases related to how they will help people living with dementia (after the workshop).	Moderate 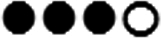
**H: Liao et al. (2022)**	BMC Geriatrics	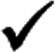	QN	Non-randomised longitudinal intervention design with control group study.	**200** *Exergaming Group*: 5 week: 40*(38)* 8 week: 40*(25)* *Companion Group*: 5 week: 41*(31)* 8 week: 42*(29)* *Control*: 37*(29)*	12−18	Intergenerational intervention with interaction via exergaming or activity companionship to improve dementia attitudes and understanding. Weekly 40 minute long sessions for either 5 or 8 weeks depending on intervention group allocation.	**Taiwan.** Nine schools. 15 day care centres.	Scores on Dementia Attitudes Scale, delivered pre-, post-, and 1, 3 and 6 months post-intervention.	Low 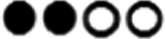
**I: Mastel-Smith et al. (2022)**	Journal of Dementia Care	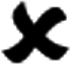	QL	One FG.	**4** *(4)*	11−19** Mean: 15.6	Online Dementia Bootcamp: virtual dementia education platform with online live sessions to improve dementia-related knowledge and perceptions. Live sessions were held once a week for six weeks and for 90 minutes per session.	**USA.** Dementia awareness club at a high school.	FG transcript descriptive analysis results.	Low 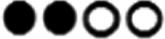
**J: Masuda et al. (2019)**	Studies in Health Technology and Informatics	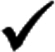	MM	Non-randomised simple within-subjects pre-/post-intervention design. Surveys.	**38**	6−12**	Pepper: Robot-mediated DEP to improve dementia awareness and knowledge. A training course of undisclosed length.	**Japan.** Elementary school students recruited through public posters.	Dementia understanding questionnaire*** score changes. Feedback survey responses.	Very Low 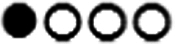
**K: Nazir et al. (2015)**	Journal of Dementia Care	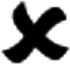	MM	Descriptive QN study. Feedback surveys.	**150**	5−19**	Dementia Awareness-Raising and Education Project: age-adapted dementia teaching sessions about dementia, and the needs of people living with dementia and their carers. Five hour-long sessions delivered over the course of a year.	**England.** Three schools.	Dementia quiz*** scores. Feedback survey responses.	Very Low 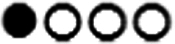
**L: Noble et al. (2015)**	Health Education and Behaviour	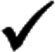	QN	Non-randomised simple within-subjects longitudinal design pilot study.	**68**	9−11	Old SCHOOL Hip-Hop: arts-based Alzheimer’s disease education programme with a mnemonic of key symptoms for improving dementia health literacy. Sessions were an hour long and delivered over three consecutive days.	**USA.** Primary school (86.4% African American students). Socio-economically disadvantaged community.	Dementia knowledge quiz*** scores, delivered at baseline, post-intervention and 3 months later.	Very Low 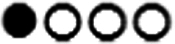
**M: Parveen et al. (2015)**	Journal of Dementia Care	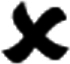	MM	Non-randomised simple within-subjects pre-/post-intervention evaluation. Feedback surveys.	**38**	14−16	Dementia Detectives: detective-themed dementia awareness session to promote advocacy, awareness and attitudes. A one-hour session.	**England.** Secondary school.	Feedback survey scores and responses.	Very Low 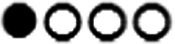
**N:** Pawlicka et al. (2023)	Health Promotion Journal of Australia	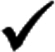	QL	40 interviews.	**40**	8−10	Project DARE: arts-based DEP to increase understanding about dementia. Three sessions delivered one day a week, over a three-week period. Each session lasted one hour.	**Australia.** Affluent primary school.	Interview transcript thematic analysis results.	Very Low 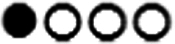
**O: Ritchie et al. (2023)**	Dementia	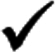	QL	Two FGs. Participatory video methods.	**23** *FG:* 20	10−11	Class in a Bag: Dementia awareness session delivered using a resource bag to improve dementia awareness and social responsibility. A single, 80 minute session.	**Scotland.** Primary school.	Results of analysis of vignettes created from 54 minutes and 36 seconds of participant-produced videos. FG transcript content analysis results.	Low 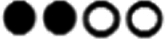
**P: Sakai and Washington University (2014)**	Thesis published by Washington University	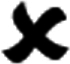	QN	Non-randomised simple within-subjects pre-/post-intervention design.	**55**	7−8	Allie Learns about Alzheimer’s Disease: Alzheimer’s disease storybook^a^ to improve knowledge, attitudes and willingness to interact with people living with dementia. 1-1.5 hour session.	**USA.** Recruitment through Washington University and elementary schools. Children with English fluency and parents with literacy at 10^th^ grade level.	Alzheimer’s disease knowledge, attitudes and patient engagement willingness questionnaire^***^ score changes. 10-item Positive and Negative Affect Scale for Children score changes. Intervention satisfaction survey scores.	Very Low 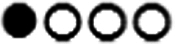
**Q: Smith et al. (2020)**	Frontiers in Public Health	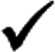	MM	Non-randomised simple within subjects pre-/post-intervention design. Undisclosed number of semi-structured interviews.	**59** *Intergenerational intervention:* 37(13) *Non-intergenerational intervention:* 22(11)	9−10	Adapted Kids4Dementia: teacher-led multimedia DEP with or without intergenerational experience to improve dementia knowledge and attitudes. One 45 minute long weekly lesson was delivered each week for 8 weeks. 45 minute long excursions began for the eligible groups from week 3 of the programme.	**Australia.** Three publicly funded primary school classes. One care service.	KIDS score changes, delivered pre-, post- and 6 months post-intervention. Interview transcript thematic analysis results (excluded).	Very Low 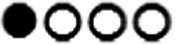

Key: DEP: Dementia Education Programme; FG: Focus Groups; MM: Mixed Methods; QL: Qualitative; QN: Quantitative. (excluded): See *Results: Review Findings* for exclusion justifications.

^*^ Shared where available.

^**^ inferred from recruitment population age range.

^***^ Bespoke measure.

^****^ Measured using GRADE or ConQual (Munn et al., 2014; Ryan & Hill, 2016). For MM studies, the level of evidence provided is whatever the lowest rating given was when GRADE and ConQual were used to assess the study’s QL and QN components (Hong et al., 2018).

### Characteristics of the included studies

Details of the 17 included studies are in Table I. The studies spanned seven different countries: England (Di Bona et al., 2019; Farina et al., 2020b, 2020a; Nazir et al., 2015; Parveen et al., 2015), Australia (Baker et al., 2019; Burns et al., 2021; Pawlicka et al., 2023; Smith et al., 2020), the United States of America (USA) (Mastel-Smith et al., 2022; Noble et al., 2015; Sakai & Washington University, 2014), Scotland (Gibson et al., 2018; Ritchie et al., 2023), Canada (Chow et al., 2018), Japan (Masuda et al., 2019), and Taiwan (Liao et al., 2022).

One of the included studies was a thesis (Sakai & Washington University, 2014); the remaining 16 were journal articles, three of which were not peer-reviewed (Mastel-Smith et al., 2022; Nazir et al., 2015; Parveen et al., 2015). Study publication dates ranged from 2014 to 2023. Study samples covered a minimum range of 7 to 18-year-olds. They included a maximum range of 5 to 19-year-olds, based on the age limits of their sampled populations when studies did not explicitly declare their age coverage. All but two recruited participants from schools alone; one used a university database (Sakai & Washington University, 2014), whilst another recruited elementary school children using publicly displayed posters (Masuda et al., 2019). The review findings represent a maximum of 1,345 children.

There were four quantitative (Farina, 2017; Liao et al., 2022; Noble et al., 2015; Sakai & Washington University, 2014), five qualitative (Farina et al., 2020a; Gibson et al., 2018; Mastel-Smith et al., 2022; Pawlicka et al., 2023; Ritchie et al., 2023), and eight mixed methods studies (Baker et al., 2019; Burns et al., 2021; Chow et al., 2018; Di Bona et al., 2019; Masuda et al., 2019; Nazir et al., 2015; Parveen et al., 2015; Smith et al., 2020). Quantitative studies had non-randomised or descriptive designs. Qualitative methods included art-based designs, focus groups, participatory video methods, interviews or self-reports. Quantitative data arose from questionnaires which measured various outcomes: dementia-related insight, knowledge and attitudes; intervention satisfaction; and confidence and motivation regarding supporting or interacting with people living with dementia. Qualitative outcome data were quotes or themes capturing participants’ experiences of or learning from interventions. Formal and informal analyses extracted these qualitative results from participant-produced artworks or videos, as well as interview, focus group or questionnaire responses.

Outcome measures were extracted from samples ranging in size between four and 301 children. The studies evaluated 15 different interventions in total. Two interventions, *Dementia Friends* (Farina et al., 2020b, 2020a) and *Project DARE* (Burns et al., 2021; Pawlicka et al., 2023), were both evaluated twice in papers which each contributed unique insights about the interventions.

The interventions studied and their aims are detailed in Table I and the Supplementary Material (Appendix 6). They included eight dementia education programmes delivered in schools by teachers, researchers or volunteers (Baker et al., 2019; Burns et al., 2021; Farina et al., 2020a, 2020b; Gibson et al., 2018; Nazir et al., 2015; Noble et al., 2015; Parveen et al., 2015; Pawlicka et al., 2023; Ritchie et al., 2023), two intergenerational programmes (Chow et al., 2018; Liao et al., 2022), one online dementia education programme (Mastel-Smith et al., 2022), one robot-mediated dementia education programme (Masuda et al., 2019), one dementia storybook reading intervention (Sakai & Washington University, 2014), and two mixed intergenerational and school-based programmes(Di Bona et al., 2019; Smith et al., 2020).

Interventions helped children better understand and support people living with dementia in diverse ways including formal teaching, interactive activities, videos, art, drama, music, meeting people living with dementia, meeting professionals or carers working with people living with dementia, dementia-themed stories, and/or experiences aiming to simulate the experience of living with dementia. The interventions’ goals and components were assessed by the authors during screening to be relevant to or potentially useful for children supporting a person living with dementia, although providing dementia care was not an explicit or exclusive focus of any intervention. The interventions’ aims mainly involved improving understanding, awareness, attitudes, literacy and advocacy regarding dementia, including in relation to improving the well-being and lives of people living with dementia.

### Review findings

**Figure 3.**
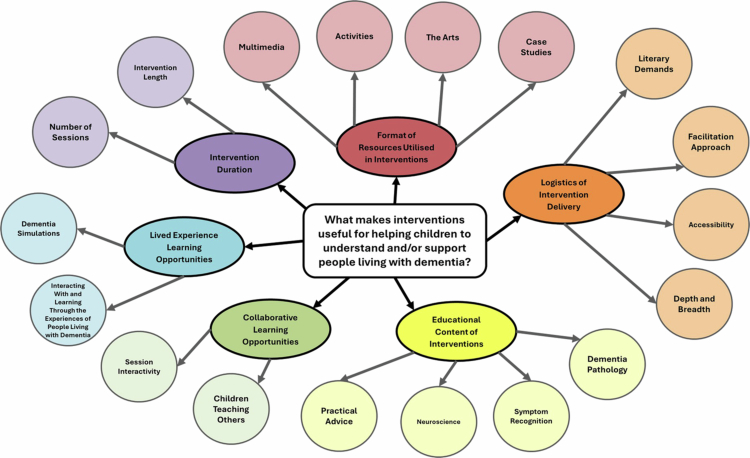
Spider diagram summarising the review findings.

Across 17 included studies, 81 review-relevant findings were extracted (Table II). Quantitative findings were excluded from one paper as they inseparably combined the results of two versions of the intervention studied (Di Bona et al., 2019). Qualitative findings were excluded from a second paper as they did not separate results discussing two versions of the studied intervention (Smith et al., 2020). The remaining qualitative findings were all eligible for inclusion in the review, as 23 were unequivocal and 36 were credible (Silver & Francis, 2022). The findings are displayed in full using their alphanumeric codes in the Supplementary Material (Appendix 3), which also details the credibility ratings for, and participant quotations supporting, qualitative findings. Figure 3 and Table III display the findings and how they were synthesised into 18 categories, which formed six integrated findings regarding what makes interventions useful for helping children understand and/or support people living with dementia. The level of evidence for each integrated finding was low.

**Table II.** Number of findings contributed from each included study.

	Number of Contributed Findings		
Qualitative Study Alpha Code(See Table I)	Qualitized	Total	Total
A	6	1	7
B	1	2	3
C	1	0	1
D	5	0	5
E	11	0	11
F	0	3	3
G	1	0	1
H	0	2	2
I	11	0	11
J	1	1	2
K	7	1	8
L	0	2	2
M	6	1	7
N	3	0	3
O	6	0	6
P	0	7	7
Q	0	2	2
Totals From All Studies	**59**	**22**	**81**

**Table III.** Visual display of the synthesis process.

Alphanumeric-Coded Qualitative (QL) and Qualitized (QZ) Findings Extracted from the Included Studies (Level of Evidence of the Study the Finding was Extracted From)	Categories	Integrated Findings *Participant Quote Exemplifying One Component Finding* (Finding Code Page of Quote in Original Study).	Integrated Finding Level of Evidence
A1(QL): Children responded positively and empathetically to animations and videos (Low)	Multimedia	**Format of Resources Utilised in Interventions** “*the videos, that made it more interesting.”* (A1, p.1784)	Low
A5(QL): Multimedia dementia education resources can improve children’s confidence with supporting people with dementia (Low)			
A7(QZ): Multimedia dementia education sessions can improve children’s attitudes relating to the personhood of people living with dementia, dementia stigma and understanding of dementia (Low)			
J1(QL): Technology malfunctioning can affect children’s experiences when learning about dementia (Very Low)			
M6(QL): Children enjoy videos when learning about dementia (Very Low)			
A2(QL): Activities were received enthusiastically and were a part that were liked best by children (Low)	Activities		
D1(QL): Completing lifestory books can help children to interact with people living with dementia (Very Low)			
I3(QL): Children enjoy learning about dementia from activities (Low)			
K3(QL): Activity-based teaching sessions can help children to understand dementia and the care role (Very Low)			
K8(QZ): Children find activity-based teaching sessions helpful for learning about dementia. (Very Low)			
M4(QL): Children like interactive and physical activities when learning about dementia (Very Low)			
O4(QL): Interventions including a mixture of diverse interactive activities can help children to learn how to support a relative living with dementia (Low)			
B1(QL): Pairing art-based and formal teaching in sessions can help children understand the experience of memory for people living with dementia as involving fading or lost memories, and disorder/chaos (Very Low)	The Arts		
B2(QZ): Pairing two art-based and one formal dementia teaching hour-long lessons can improve children’s understanding of people living with dementia (Very Low)			
B3(QZ): Pairing two art-based and one formal dementia teaching hour-long lessons did not improve children’s confidence with interacting with people living with dementia (Very Low)			
K2(QL): Roleplay can be an enjoyable way for children to learn about dementia (Very Low)			
L1(QZ): Creative arts-based interventions can improve children’s recognition and neuroscientific understanding of Alzheimer’s disease symptoms (Very Low)			
N1(QL): Pairing art-based and formal teaching in sessions can help children understand the challenges, memory symptoms, and frustrations related to dementia (Very Low)			
N2(QL): Pairing art-based and formal teaching in sessions can help children develop resilience and positive coping strategies related to dementia (Very Low)			
N3(QL): Children find art a useful and enjoyable medium for expressing their thoughts, feelings and learning about dementia.			
K7(QL): Children find case studies of celebrities living with dementia interesting (Very Low)	Case Studies		
P1(QZ): Reading an Alzheimer’s disease-related storybook can improve attitudes and objective and self-reported knowledge about the condition. (Very Low)			
P2(QZ): Reading and discussing Alzheimer’s disease related storybooks does not seem to affect the distress levels of children (Very Low)			
P4(QZ): The impact reading and discussing storybooks related to Alzheimer’s disease had on children’s willingness to support individuals with Alzheimer’s disease was variable and stronger for behaviours related to spending time with people with the condition than those related to physically supporting them (Very Low)			
P5(QZ): Most children like reading storybooks related to Alzheimer’s disease (Very Low)			
D2(QL): Completing lifestory books are not preferable for all children due to some preferring spontaneous listening and chatting or disliking the literary demands (Very Low)	Literary Demands	**Logistics of Intervention Delivery** “*[facilitators] are really like nice and trying to engage everyone.”* (E4, p.352)	Low
I11(QL): Children can dislike having too much independent work and reading and prefer learning through presentations (Low)			
L2(QZ): Children retain information about Alzheimer’s symptoms for longer when it is also included in a memory aid like a mnemonic or song (Very Low)			
E4(QL): Children benefit from education interventions being delivered positively (Moderate)	Facilitation Approach		
I5(QL): Virtual teaching from and meeting healthcare professionals, dementia patients and dementia carers can help children to feel confident with supporting a person living with dementia (Low)			
I6(QL): Virtual teaching from and meeting healthcare professionals, dementia patients and dementia carers can help children understand and empathise with the personhood of a person living with dementia (Low)			
J2(QZ): Learning about dementia through a humanoid robot simulating and teaching about dementia can help children to understand and improve their knowledge about dementia (Very Low)			
P7(QZ): Where parents lead storybook reading and discussion interventions, the parent having higher education and previous dementia work experiences can be negatively associated with children’s attitudes and willingness to engage with people with Alzheimer’s disease post-intervention (Very Low)			
Q1(QZ): School-based dementia teaching over the course of two months with or without an intergenerational experience can result in prolonged objective improvements in children’s dementia attitudes and knowledge (Very Low)			
E10(QL): Children believe it would be useful for dementia education interventions for young people to be freely accessible (Moderate)	Accessibility		
I2(QL): Online learning management systems for hosting dementia education content are helpful (Low)			
E7(QL): Children appreciate interventions providing in-depth factual detail about dementia, including its different types, causes and symptoms (Moderate)	Depth and Breadth		
K5(QL): Children wish to understand the research evidence base around dementia from interventions (Very Low)			
P3(QZ): Reading and discussing storybooks related to Alzheimer’s disease does not clear up misunderstandings of all dementia facts, particularly when not explicitly shared in the book (Very Low)			
A3(QL): Learning about the occurrence of younger-onset dementia and the non-contagiousness of the condition stood out to children (Low)	Dementia Pathology	Educational Content of Interventions “*there’s just so many different aspects of the disease that could be delved into.”* (I7, Discussion)	Low
D5(QL): Interventions need to provide children with certainty about dementia’s cause (Very Low)			
I7(QL): Students felt it was important to learn about the disease and brain elements of dementia (Low)			
M1(QL): Children enjoy learning about types of dementia (Very Low)			
O3(QL): Including information about dementia risk factors can create worry for some children regarding their own dementia risk (Low)			
I8(QL): Children benefit from learning about how emotions in dementia can be separate to memories (Low)	Symptom Recognition		
K6(QL): Children consider practising diagnosing patients with dementia using standardised examinations (Very Low)			
A4(QL): Children enjoyed and benefitted from understanding dementia’s impact on different interests and functions through being taught about the brain (Low)	Neuroscience		
K1(QL): Children find brain models helpful for understanding dementia (Very Low)			
O1(QL): Visual models can help children to understand the neuroscience of dementia (Low)			
E9(QL): Children appreciate interventions providing practical ways of helping, identifying and engaging with people living with dementia (Moderate)	Practical Advice		
M3(QL): Children enjoy discovering practical ways they can help someone with dementia through joining dementia championing schemes (Very Low)			
O2(QL): Sharing examples of assistive technology can help children to understand and adapt support strategies for people living with dementia (Low)			
A6(QL): Children respond positively to opportunities to teach others about dementia (Low)	Children Teaching Others	Collaborative Learning Opportunities*“it’s cool to talk to other people who are learning at the same time as you.”* (I9, Students’ responses)	Low
K4(QL): Children reflect on opportunities to teach others following dementia education (Very Low)			
O5(QL): Children reflect on opportunities to educate their parents about their dementia education (Low)			
E5(QL): Children benefit from interventions being interactive (Moderate)	Session Interactivity		
G1(QL): Interactive sessions can help children to learn that they can support people with dementia through socially interacting with them, offering assistance and communicating appropriately with them (Moderate)			
I9(QL): Children enjoy opportunities to interact with peers when learning about dementia (Low)			
M2(QL): Children enjoy group discussions of dementia (Very Low)			
P6(QZ): Not all children enjoy formally discussing Alzheimer’s disease-related storybooks when asked questions about it by a parent (Very Low)			
C1(QL): Having quality visits with people living with dementia can help children become more insightful about and understanding of people with dementia (Very Low)	Interacting With and Learning Through the Experiences of People Living with Dementia	**Lived Experience Learning Opportunities** “*imagining what it would be like to like, live with a person with dementia.”* (N6, p.486)	Low
D3(QL): Visiting people with dementia can help children to learn about how to support them (Very Low)			
D4(QL): Visiting people with dementia can help children to relate to and understand people living with dementia (Very Low)			
E8(QL): Children appreciate interventions that involve learning from people with dementia (Moderate)			
H1(QZ): Intergenerational interactions with people living with dementia, via either playing physical exercise-utilising video games or accompanying them during their daily activities, can significantly improve adolescents’ social comfort and knowledge about dementia (Low)			
I1(QL): Children feel interacting with people living with dementia can help them to understand people with the condition (Low)			
M5(QL): Children like to meet people living with dementia face to face to learn about the experience of living with dementia (Very Low)			
I4(QL): Virtual dementia simulations can help children to understand dementia (Low)	Dementia Simulations		
O6(QL): Simulation and sensory experiences, particularly with scent boxes and goggles modified to reflect vision in dementia, can help children to understand and discover how to alleviate the non-memory related symptoms of dementia (Low)			
F1(QZ): A single dementia education session does not objectively, but does subjectively, improve understanding of people living with dementia (Low)	Number of Sessions	**Intervention Duration** “*maybe if we had like two more sessions in it or something.”* (E11, p.352)	Low
F2(QZ): Children enjoy single dementia education sessions (Low)			
F3(QZ): A single dementia education session does not improve behavioural intentions to support individuals living with dementia by working professionally with them (Low)			
E1(QL): A single dementia education session can give children new understandings about dementia in terms of its different types, symptoms and experiences of people living with dementia (Moderate)			
E2(QL): A single dementia education session can give children new understandings about dementia in relation to their perceptions of people living with dementia and empowered them to support these people (Moderate)			
E3(QL): A single dementia education session can improve children’s intentions to spend time with and work with people with dementia in the future (Moderate)			
E11(QL): Children desired more than one dementia education session (Moderate)			
M7(QZ): A single-session dementia awareness programme is enjoyable, understandable and can improve children’s perceived knowledge of dementia (Very Low)			
E6(QL): Children believed around an hour was appropriate for a dementia education session (Moderate)	Intervention Length		
H2(QZ): Weekly intergenerational interventions need to run for more than 5 weeks for meaningful changes in knowledge of and social comfort towards dementia to be detected (Low)			
I10(QL): Children prefer to be allocated a longer time period for sessions of around 2 hours which could be terminated early when learning about dementia (Low)			
Q2(QZ): Intergenerational experiences do not augment the objective improvements to children’s dementia attitudes and knowledge obtained by receiving just school-based dementia teaching over the course of 2 months (Very Low)			

## Integrated findings

### Integrated finding 1: format of resources utilised in interventions

Twenty-five findings (16 qualitative and nine qualitized) from ten studies made the first integrated finding. This reports the types of resources included in child-targeted dementia interventions which contributed to intervention usefulness. There were four categories of useful resources: multimedia, activities, the arts and case studies.

#### Category 1: multimedia

Improvements in children’s dementia attitudes and understandings came from interventions utilising various media types, with specific endorsements received for animations and videos. However, technology should be used cautiously since children identified that malfunctions impair their learning.

#### Category 2: activities

Children enjoyed activities, which also helped them to understand and learn about dementia and its care effectively. Diverse, interactive and physical activities were endorsed.

#### Category 3: the arts

Children relish learning about dementia through arts-based strategies, including drama and artwork. These appeared to be effective ways for helping children to understand the more complex facets of dementia, including its symptoms and presentation, but were not always effective for helping children develop confidence interacting with people living with dementia.

#### Category 4: case studies

Children appreciated learning about dementia through fictional case studies described in storybooks. They also reported finding real-life examples of celebrities living with dementia intriguing. Reading storybooks was useful for improving children’s attitudes and knowledge about dementia without causing them significant emotional distress. Storybooks also impacted children’s willingness to support individuals living with Alzheimer’s disease, but more so in terms of spending time with them instead of physically supporting them.

### Integrated finding 2: logistics of intervention delivery

The second integrated finding was constructed from 14 findings (eight qualitative and five qualitized) from eight studies. It highlighted that the usefulness of interventions depended on their structure and management. There were four things to consider to usefully organise interventions: intervention literacy demands, how interventions were facilitated, intervention accessibility, and the depth and breadth of intervention content.

#### Category 5: literacy demands

Interventions must be mindful of involving too much reading and writing since children expressed dislike for overuse of these formats. Preferred structures included spontaneous listening, chatting and presentations. Dementia symptom information was also retained better by children when structured using fewer literacy demands, such as within songs or mnemonics.

#### Category 6: facilitation approach

Children found interventions useful when they were facilitated appropriately by leaders. This included intervention leaders being enthusiastic and positive. There was inferential evidence that having previous dementia work experience may hinder facilitators in providing this optimism, which could impact how useful interventions are for changing children’s attitudes, particularly regarding supporting people living with dementia (Sakai & Washington University, 2014). Children responded positively to various facilitator types, including professionals, patients, carers and robots. Virtual and in-person session facilitation both appeared to support children’s learning.

#### Category 7: accessibility

Children recommend that interventions should be freely and easily accessible. One organisation system referenced as promoting this was online learning management systems.

#### Category 8: depth and breadth

Learning about dementia seemed to be facilitated by interventions being structured to provide a high level of detail about the condition. Facts about dementia which were not explicitly shared during interventions were not always well understood afterwards. Further, children appeared to find depth and breadth useful since they appreciated learning about complex aspects of dementia, including its research evidence base.

### Integrated finding 3: educational content of interventions

The third integrated finding synthesised 13 qualitative findings from seven studies. The finding showed the dementia education content of interventions can dictate how useful children find them. There were four categories of content which children particularly valued: dementia pathology, symptom recognition, neuroscience and practical advice relating to dementia care.

#### Category 9: dementia pathology

Children highlighted that it was beneficial and enjoyable for them to learn about the causes of dementia, particularly its non-contagiousness, different onset ages and types, and brain origin. However, sharing dementia risk factors with children must be done sensitively, as this created worry for some children regarding their own dementia risk.

#### Category 10: symptom recognition

Including information about discriminating dementia symptoms engaged children, demonstrated by their desire to use standardised examinations to practise diagnosing the condition. Children found it particularly useful to understand how emotions can be distinct from dementia memory symptoms, perhaps as it helped them to process dementia in a loved one by recognising there was maintenance of some of their character.

#### Category 11: neuroscience

Children enjoyed and seemed to understand dementia’s impact better through being educated about the brain. Visual brain models were recognised as useful tools for providing this teaching.

#### Category 12: practical advice

Interventions must endeavour to recommend children practical ways of supporting and recognising people living with dementia, as children value this material. Useful ways of providing this content included signposting children to further education and involvement opportunities, as well as exemplifying assistive technology relevant to people living with dementia.

### Integrated finding 4: collaborative learning opportunities

Eight findings (seven qualitative and one qualitized) from eight studies formed the fourth integrated finding. It highlighted that children find interventions which offer opportunities for collaborative learning useful. There were two categories of helpful ways that such education could be achieved: children teaching others, and session interactivity.

#### Category 13: children teaching others

Teaching others, such as parents, about dementia was enjoyable and memorable for children, suggesting these opportunities contribute to the usefulness of dementia interventions.

#### Category 14: session interactivity

Children’s learning from and enjoyment of interventions was enhanced when intervention sessions were interactive. Notable ways of achieving this included providing opportunities for peer or group discussions. Children, however, did not always find it enjoyable to discuss dementia formally one-on-one with a parent, meaning interactivity within larger groups or with fellow children may be preferred.

### Integrated finding 5: lived experience learning opportunities

The fifth integrated finding comprised ten findings (eight qualitative and two qualitized) from eight studies. It showed children found direct experiences of dementia useful for improving their confidence with understanding and supporting people living with dementia. There were two categories of experiential learning opportunities: interacting with and learning through the experiences of people living with dementia, and dementia simulations.

#### Category 15: interacting with and learning through the experiences of people living with dementia

Children enjoyed engaging with people living with dementia in person, including through visiting, gaming and completing activities with them. These opportunities subjectively and objectively improved children’s knowledge about dementia and their confidence regarding being exposed to and supporting people living with dementia. Whilst such learning was achievable through school- based dementia teaching alone without intergenerational experiences, children vehemently appraised these first-hand dementia exposures as useful and insightful.

#### Category 16: dementia simulations

Interventions which allow children to experience dementia symptoms for themselves were useful for helping children understand dementia and its memory-related symptoms. Children identified helpful in-person simulation activities as those which replicated dementia symptoms. For example, one activity in Ritchie et al. (2023) aimed to imitate the frustration and confusion experienced by some people living with dementia by giving children a simple task to complete whilst listening to white noise, donning gloves, and wearing modified goggles with obscured lenses. Virtual simulations of dementia were also deemed beneficial.

### Integrated finding 6: intervention duration

The final integrated finding collated 12 findings (six qualitative and six quantitative) from six studies to highlight how the duration of interventions was integral to their usefulness. There were two categories of helpful time scale considerations: the number of sessions and intervention length.

#### Category 17: number of sessions

Children appraised single-session interventions as helping them to understand dementia better and empowering them to support people living with dementia. However, after a single session children did not objectively show significant improvements in their behavioural intentions to support people living with dementia professionally. This suggests that it may be more useful for dementia interventions to include multiple sessions, which children also reported as beneficial.

#### Category 18: intervention length

Children believed an hour was an appropriate amount of time for a dementia education session to be useful. They acknowledged, however, that it may be more tactful to schedule for more time, potentially two hours, due to preferring early termination of sessions over overrunning. Where interventions involved intergenerational experiences alone, they were only useful for objectively changing dementia understanding and social comfort when they ran for over five weeks. When two months long, school-based dementia education interventions with or without intergenerational experiences both produced similar objective improvements in children’s dementia attitudes and knowledge.

## Discussion

This is the first systematic review exploring the usefulness of delivered dementia care-relevant interventions for children. The review synthesises 17 studies evaluating 15 diverse interventions, from seven countries, and demonstrates six integrated findings regarding what makes interventions useful for helping children to understand and/or support people living with dementia (Tables I and III). The review represents a sample of 1,345 participants from various countries, ethnicities, and socioeconomic statuses (Table I).

The findings offer suggestions for designing interventions for children and young people needing support with navigating a dementia diagnosis for someone they care for. For adult informal caregivers of people living with dementia, interventions commonly use psychoeducation and therapy principles, and the majority seem to be delivered online or via telephone or video (Encinas-Monge et al., 2024). Child learning theories suggest children’s learning is supported by exploration, play and scaffolding more than for adults (Liquin & Gopnik, 2022; Piaget, 1999; Vygotsky, 1984), and our findings suggest a wider variety of resources, activities, facilitation approaches, interactivity, content and delivery methods may be beneficial for children receiving dementia-care related interventions. An accessible online learning portal could host virtual elements of the intervention, potentially including learning modules about the neuroscience, symptoms and pathology of dementia and practical advice for supporting people living with dementia. The modules could include multimedia, especially videos, case studies, art-based learning, and activities and offer experiential learning through dementia simulations, as in Mastel-Smith et al. (2021). Further, opportunities to learn from or interact with people living with dementia are recommended, perhaps by including videos of real or fictional people living with dementia.

Live intervention sessions may help consolidate virtual learning by giving children opportunities to discuss and teach their understanding with peers. Live sessions could clarify misunderstandings and alleviate any worries caused by materials. The findings also suggest that the intervention should be facilitated enthusiastically and positively, have appropriate literacy demands, and provide a high level of detail about dementia. Through summarising the findings regarding intervention duration, such interventions are also suggested to be 1-2 hours long and delivered over at least six weeks for most impact.

## Limitations

All the included studies are considered low quality, thus evidence should be applied cautiously. It was difficult to conclude exactly which intervention elements determined intervention usefulness because they had diverse components each not explicitly evaluated. The lack of translation may have limited the review’s cultural reach by excluding papers not written in English. This should be sought in future studies to provide insight into designing interventions for the UK’s multicultural population (Ashcroft & Bevir, 2018). Children with additional needs or learning disabilities, and children experiencing digital poverty, were also not explicitly researched. Future work could therefore explore how to appropriately personalise and grade interventions for unexplored groups to help more children access support.

A strength of the review was that by excluding evidence not collected by children directly, it aligns with recommended clinical practice to design interventions for young people with young people (Larsson et al., 2018). Following this philosophy means this work has provided researchers with helpful insight into what may work for children to inspire provisional intervention designs. These helpful initial indicators will better equip researchers to consult the target population on what would make a child-targeted intervention useful. Co-design conducted with this in mind is necessary as an intervention’s target population will likely be young carers, whereas the included studies generally sampled typical schoolchildren without differentiating their level of personal experiences with dementia. As more research become available, conducting another search with a narrower focus on what would benefit young carers specifically in dementia-care related interventions could also be explored.

This comprehensive review included a range of studies, including grey literature, which is important given the limited evidence base exploring this topic. However, inclusion of non-peer-reviewed studies and studies assessed as low or very-low quality may be limitations of this review. It is recommended future reviews consider only including robust, high-quality peer-reviewed studies and look for the potential to conduct a robust quantitative analysis. We made a pragmatic decision to include studies on child-targeted interventions which included a subset of young adults due to interventions being delivered in schools, to provide a comprehensive review. Three studies (354 participants) may have incorporated results from 18-19-year-olds (Liao et al., 2022; Mastel-Smith et al., 2022; Nazir et al., 2015), thus limiting the generalisability of some findings to younger children. Future research should explore and evaluate the impact of tailoring interventions for children at different stages of development, who likely have different literacy skills and learning preferences.

## Conclusion

This comprehensive review provides novel insights into the logistics of delivery, duration, content and format of resources that may be used to support children understand and/or support people living with dementia. Our work is a pivotal step in supporting young carers of people living with dementia, since the disruption of providing dementia care without appropriate support on children’s health and wellbeing has been evidenced (Cartwright et al., 2021; Gelman & Rhames, 2018). These challenges are likely to increase due to the ageing population. Our recommendations could inform interventions for children with exposure to dementia care. These initial indicators will better equip researchers to consult the target population on what would make a child-targeted intervention useful. Co-design is anticipated to be necessary as the target population for the intervention may include young carers, whereas the included studies generally sampled typical schoolchildren. Further work should avoid relying on these recommendations alone due to the low-quality of evidence. Future research should rigorously evaluate child-targeted dementia care-relevant interventions and dissect their usefulness more precisely, unravelling the most beneficial intervention components for different demographics of children.

## Supplementary Material

Supplementary Material Supplementary_Material_TF.docx

## Data Availability

The authors confirm that the data supporting the findings of this study are available within the article and its supplementary materials.
