# Supplementary material for: Interventions to support young carers/supporters of people living with dementia: a mixed methods systematic review
Source: Int J Qual Stud Health Well-being. 2026 Mar 27;21(1):2650367. doi: 10.1080/17482631.2026.2650367 (PMC13034709; doi:10.1080/17482631.2026.2650367)
Supplement: Supplementary Material — Supplementary_Material_TF.docx [file ZQHW_A_2650367_SM6463.docx]

APPENDIX 1: PRISMA 2020 CHECKLIST

| **Section and Topic** | **Item #** | **Checklist item** | **Location where item is reported** |
| --- | --- | --- | --- |
| **TITLE** | | |  |
| Title | 1 | Identify the report as a systematic review. | Title and Methods |
| **ABSTRACT** | | |  |
| Abstract | 2 | See the PRISMA 2020 for Abstracts checklist. | Abstract |
| **INTRODUCTION** | | |  |
| Rationale | 3 | Describe the rationale for the review in the context of existing knowledge. | Introduction |
| Objectives | 4 | Provide an explicit statement of the objective(s) or question(s) the review addresses. | Introduction |
| **METHODS** | | |  |
| Eligibility criteria | 5 | Specify the inclusion and exclusion criteria for the review and how studies were grouped for the syntheses. | Methods |
| Information sources | 6 | Specify all databases, registers, websites, organisations, reference lists and other sources searched or consulted to identify studies. Specify the date when each source was last searched or consulted. | Methods |
| Search strategy | 7 | Present the full search strategies for all databases, registers and websites, including any filters and limits used. | Supplementary Material |
| Selection process | 8 | Specify the methods used to decide whether a study met the inclusion criteria of the review, including how many reviewers screened each record and each report retrieved, whether they worked independently, and if applicable, details of automation tools used in the process. | Methods |
| Data collection process | 9 | Specify the methods used to collect data from reports, including how many reviewers collected data from each report, whether they worked independently, any processes for obtaining or confirming data from study investigators, and if applicable, details of automation tools used in the process. | Methods |
| Data items | 10a | List and define all outcomes for which data were sought. Specify whether all results that were compatible with each outcome domain in each study were sought (e.g. for all measures, time points, analyses), and if not, the methods used to decide which results to collect. | Methods |
|  | 10b | List and define all other variables for which data were sought (e.g. participant and intervention characteristics, funding sources). Describe any assumptions made about any missing or unclear information. | Methods |
| Study risk of bias assessment | 11 | Specify the methods used to assess risk of bias in the included studies, including details of the tool(s) used, how many reviewers assessed each study and whether they worked independently, and if applicable, details of automation tools used in the process. | Methods |
| Effect measures | 12 | Specify for each outcome the effect measure(s) (e.g. risk ratio, mean difference) used in the synthesis or presentation of results. | Methods |
| Synthesis methods | 13a | Describe the processes used to decide which studies were eligible for each synthesis (e.g. tabulating the study intervention characteristics and comparing against the planned groups for each synthesis (item #5)). | Methods |
|  | 13b | Describe any methods required to prepare the data for presentation or synthesis, such as handling of missing summary statistics, or data conversions. | Methods |
|  | 13c | Describe any methods used to tabulate or visually display results of individual studies and syntheses. | Methods |
|  | 13d | Describe any methods used to synthesize results and provide a rationale for the choice(s). If meta-analysis was performed, describe the model(s), method(s) to identify the presence and extent of statistical heterogeneity, and software package(s) used. | Methods |
|  | 13e | Describe any methods used to explore possible causes of heterogeneity among study results (e.g. subgroup analysis, meta-regression). | N/A - not relevant for the synthesis and data used |
|  | 13f | Describe any sensitivity analyses conducted to assess robustness of the synthesized results. | N/A - not relevant for the synthesis used. |
| Reporting bias assessment | 14 | Describe any methods used to assess risk of bias due to missing results in a synthesis (arising from reporting biases). | N/A due to review design |
| Certainty assessment | 15 | Describe any methods used to assess certainty (or confidence) in the body of evidence for an outcome. | Methods |
| **RESULTS** | | |  |
| Study selection | 16a | Describe the results of the search and selection process, from the number of records identified in the search to the number of studies included in the review, ideally using a flow diagram. | Results |
|  | 16b | Cite studies that might appear to meet the inclusion criteria, but which were excluded, and explain why they were excluded. | Supplementary Material |
| Study characteristics | 17 | Cite each included study and present its characteristics. | Results - Table |
| Risk of bias in studies | 18 | Present assessments of risk of bias for each included study. | Supplementary Material |
| Results of individual studies | 19 | For all outcomes, present, for each study: (a) summary statistics for each group (where appropriate) and (b) an effect estimate and its precision (e.g. confidence/credible interval), ideally using structured tables or plots. | N/A due to data collected |
| Results of syntheses | 20a | For each synthesis, briefly summarise the characteristics and risk of bias among contributing studies. | Results |
|  | 20b | Present results of all statistical syntheses conducted. If meta-analysis was done, present for each the summary estimate and its precision (e.g. confidence/credible interval) and measures of statistical heterogeneity. If comparing groups, describe the direction of the effect. | N/A due to synthesis method used |
|  | 20c | Present results of all investigations of possible causes of heterogeneity among study results. | N/A due to data collected |
|  | 20d | Present results of all sensitivity analyses conducted to assess the robustness of the synthesized results. | N/A due to synthesis method used |
| Reporting biases | 21 | Present assessments of risk of bias due to missing results (arising from reporting biases) for each synthesis assessed. | N/A due to review design |
| Certainty of evidence | 22 | Present assessments of certainty (or confidence) in the body of evidence for each outcome assessed. | Supplementary Material |
| **DISCUSSION** | | |  |
| Discussion | 23a | Provide a general interpretation of the results in the context of other evidence. | Discussion |
|  | 23b | Discuss any limitations of the evidence included in the review. | Discussion |
|  | 23c | Discuss any limitations of the review processes used. | Discussion |
|  | 23d | Discuss implications of the results for practice, policy, and future research. | Discussion |
| **OTHER INFORMATION** | | |  |
| Registration and protocol | 24a | Provide registration information for the review, including register name and registration number, or state that the review was not registered. | Methods |
|  | 24b | Indicate where the review protocol can be accessed, or state that a protocol was not prepared. | Methods |
|  | 24c | Describe and explain any amendments to information provided at registration or in the protocol. | N/A - protocol adhered to |
| Support | 25 | Describe sources of financial or non-financial support for the review, and the role of the funders or sponsors in the review. | Funding |
| Competing interests | 26 | Declare any competing interests of review authors. | Disclosure statement. |
| Availability of data, code and other materials | 27 | Report which of the following are publicly available and where they can be found: template data collection forms; data extracted from included studies; data used for all analyses; analytic code; any other materials used in the review. | Supplementary Materials. |

*From:*  Page MJ, McKenzie JE, Bossuyt PM, Boutron I, Hoffmann TC, Mulrow CD, et al. The PRISMA 2020 statement: an updated guideline for reporting systematic reviews. BMJ 2021;372:n71. doi: 10.1136/bmj.n71

For more information, visit: <http://www.prisma-statement.org/>

APPENDIX 2: Line-By-Line Database Search Strategies and Results

| **EBSCOhost CINAHL Ultimate Search Strategy and Results (Searched on 9^th^ February 2024)** | | |
| --- | --- | --- |
| **Search Options** | | |
| **Limiters** - Date of Publication: 20130101-; English Language.  **Search modes** - Boolean/Phrase. | | |
| **Search ID** | **Search Terms** | **Results** |
| S13 | S9 AND S10 AND S11 AND S12 | 984 |
| S12 | S7 OR S8 | 60,432 |
| S11 | S5 OR S6 | 2,818,675 |
| S10 | S3 OR S4 | 2,226,830 |
| S9 | S1 OR S2 | 569,858 |
| S8 | TI dementia OR Alzheimer* | 39,839 |
| S7 | AB dementia OR Alzheimer* | 47,793 |
| S6 | AB opinion* OR attitude* OR belie* OR perce* OR feel* OR felt OR knowledge OR underst* OR aware* OR educat* OR impact* OR improve* OR reduc* OR confiden* OR mood OR analys* OR outcome* OR useful* OR benefi* OR disadvantag* OR efficacy OR advantag* OR evaluat* OR strength* OR weakness* OR limit* OR learn* OR effect* OR affect* OR esteem OR abilit* OR qualit* | 2,364,529 |
| S5 | TI opinion* OR attitude* OR belie* OR perce* OR feel* OR felt OR knowledge OR underst* OR aware* OR educat* OR impact* OR improve* OR reduc* OR confiden* OR mood OR analys* OR outcome* OR useful* OR benefi* OR disadvantag* OR efficacy OR advantag* OR evaluat* OR strength* OR weakness* OR limit* OR learn* OR effect* OR affect* OR esteem OR abilit* OR qualit* | 1,287,103 |
| S4 | TI intervention* OR initiative* OR scheme* OR manage* OR strateg* OR program* OR educat* OR module* OR teach* OR taught* OR course* OR group* OR activit* OR project* OR resource* OR therap* OR support* OR help* OR advice OR class* OR lesson* | 751,206 |
| S3 | AB intervention* OR initiative* OR scheme* OR manage* OR strateg* OR program* OR educat* OR module* OR teach* OR taught* OR course* OR group* OR activit* OR project* OR resource* OR therap* OR support* OR help* OR advice OR class* OR lesson* | 1,929,957 |
| S2 | AB child* OR "young pe*" OR "young care*" OR youth OR teen* OR adolescen* OR grandchild* OR grand-child* OR school* OR preschool* OR pre-school* OR paed* OR pedia* OR kid OR kids | 445,617 |
| S1 | TI child* OR "young pe*" OR "young care*" OR youth OR teen* OR adolescen* OR grandchild* OR grand-child* OR school* OR preschool* OR pre-school* OR paed* OR pedia* OR kid OR kids | 373,596 |

| **EBSCOhost MEDLINE Ultimate Search Strategy and Results (Searched on 9^th^ February 2024)** | | |
| --- | --- | --- |
| **Search Options** | | |
| **Limiters** - Date of Publication: 20130101-; English Language.  **Search modes** - Boolean/Phrase. | | |
| **Search ID** | **Search Terms** | **Results** |
| S13 | S9 AND S10 AND S11 AND S12 | 2,106 |
| S12 | S7 OR S8 | 167,983 |
| S11 | S5 OR S6 | 10,843,438 |
| S10 | S3 OR S4 | 8,092,126 |
| S9 | S1 OR S2 | 1,069,466 |
| S8 | TI dementia OR Alzheimer* | 88,561 |
| S7 | AB dementia OR Alzheimer* | 152,101 |
| S6 | AB opinion* OR attitude* OR belie* OR perce* OR feel* OR felt OR knowledge OR underst* OR aware* OR educat* OR impact* OR improve* OR reduc* OR confiden* OR mood OR analys* OR outcome* OR useful* OR benefi* OR disadvantag* OR efficacy OR advantag* OR evaluat* OR strength* OR weakness* OR limit* OR learn* OR effect* OR affect* OR esteem OR abilit* OR qualit* | 10,432,576 |
| S5 | TI opinion* OR attitude* OR belie* OR perce* OR feel* OR felt OR knowledge OR underst* OR aware* OR educat* OR impact* OR improve* OR reduc* OR confiden* OR mood OR analys* OR outcome* OR useful* OR benefi* OR disadvantag* OR efficacy OR advantag* OR evaluat* OR strength* OR weakness* OR limit* OR learn* OR effect* OR affect* OR esteem OR abilit* OR qualit* | 3,524,651 |
| S4 | TI intervention* OR initiative* OR scheme* OR manage* OR strateg* OR program* OR educat* OR module* OR teach* OR taught* OR course* OR group* OR activit* OR project* OR resource* OR therap* OR support* OR help* OR advice OR class* OR lesson* | 1,744,419 |
| S3 | AB intervention* OR initiative* OR scheme* OR manage* OR strateg* OR program* OR educat* OR module* OR teach* OR taught* OR course* OR group* OR activit* OR project* OR resource* OR therap* OR support* OR help* OR advice OR class* OR lesson* | 7,773,716 |
| S2 | AB child* OR "young pe*" OR "young care*" OR youth OR teen* OR adolescen* OR grandchild* OR grand-child* OR school* OR preschool* OR pre-school* OR paed* OR pedia* OR kid OR kids | 970,088 |
| S1 | TI child* OR "young pe*" OR "young care*" OR youth OR teen* OR adolescen* OR grandchild* OR grand-child* OR school* OR preschool* OR pre-school* OR paed* OR pedia* OR kid OR kids | 624,723 |

| **EBSCOhost APA PsycINFO Search Strategy and Results (Searched on 9^th^ February 2024)** | | |
| --- | --- | --- |
| **Search Options** | | |
| **Limiters** - Date of Publication: 20130101-; English Language.  **Search modes** - Boolean/Phrase. | | |
| **Search ID** | **Search Terms** | **Results** |
| S13 | S9 AND S10 AND S11 AND S12 | 1,169 |
| S12 | S7 OR S8 | 57,177 |
| S11 | S5 OR S6 | 1,821,986 |
| S10 | S3 OR S4 | 1,468,935 |
| S9 | S1 OR S2 | 479,564 |
| S8 | TI dementia OR Alzheimer* | 33,149 |
| S7 | AB dementia OR Alzheimer* | 56,433 |
| S6 | AB opinion* OR attitude* OR belie* OR perce* OR feel* OR felt OR knowledge OR underst* OR aware* OR educat* OR impact* OR improve* OR reduc* OR confiden* OR mood OR analys* OR outcome* OR useful* OR benefi* OR disadvantag* OR efficacy OR advantag* OR evaluat* OR strength* OR weakness* OR limit* OR learn* OR effect* OR affect* OR esteem OR abilit* OR qualit* | 1,809,754 |
| S5 | TI opinion* OR attitude* OR belie* OR perce* OR feel* OR felt OR knowledge OR underst* OR aware* OR educat* OR impact* OR improve* OR reduc* OR confiden* OR mood OR analys* OR outcome* OR useful* OR benefi* OR disadvantag* OR efficacy OR advantag* OR evaluat* OR strength* OR weakness* OR limit* OR learn* OR effect* OR affect* OR esteem OR abilit* OR qualit* | 684,303 |
| S4 | TI intervention* OR initiative* OR scheme* OR manage* OR strateg* OR program* OR educat* OR module* OR teach* OR taught* OR course* OR group* OR activit* OR project* OR resource* OR therap* OR support* OR help* OR advice OR class* OR lesson* | 399,323 |
| S3 | AB intervention* OR initiative* OR scheme* OR manage* OR strateg* OR program* OR educat* OR module* OR teach* OR taught* OR course* OR group* OR activit* OR project* OR resource* OR therap* OR support* OR help* OR advice OR class* OR lesson* | 1,458,026 |
| S2 | AB child* OR "young pe*" OR "young care*" OR youth OR teen* OR adolescen* OR grandchild* OR grand-child* OR school* OR preschool* OR pre-school* OR paed* OR pedia* OR kid OR kids | 472,762 |
| S1 | TI child* OR "young pe*" OR "young care*" OR youth OR teen* OR adolescen* OR grandchild* OR grand-child* OR school* OR preschool* OR pre-school* OR paed* OR pedia* OR kid OR kids | 273,228 |

| **WebofScience Search Strategy and Results (Searched on 9^th^ February 2024)** | | |
| --- | --- | --- |
| **Search Options** | | |
| **Timespan:** 2013-01-01 to 2024-02-09  **Limiters** - English Language  **Search modes** – Exact Search | | |
| **Search #** | **Search Terms** | **Results** |
| 13 | #9 AND #10 AND #11 AND #12 | 1,963 |
| 12 | #7 OR #8 | 177,031 |
| 11 | #5 OR #6 | 22,708,794 |
| 10 | #3 OR #4 | 15,071,767 |
| 9 | #1 OR #2 | 1,551,877 |
| 8 | TI=(dementia OR Alzheimer*) | 97,951 |
| 7 | AB=(dementia OR Alzheimer*) | 151,531 |
| 6 | AB=(opinion* OR attitude* OR belie* OR perce* OR feel* OR felt OR knowledge OR underst* OR aware* OR educat* OR impact* OR improve* OR reduc* OR confiden* OR mood OR analys* OR outcome* OR useful* OR benefi* OR disadvantag* OR efficacy OR advantag* OR evaluat* OR strength* OR weakness* OR limit* OR learn* OR effect* OR affect* OR esteem OR abilit* OR qualit*) | 21,112,454 |
| 5 | TI=(opinion* OR attitude* OR belie* OR perce* OR feel* OR felt OR knowledge OR underst* OR aware* OR educat* OR impact* OR improve* OR reduc* OR confiden* OR mood OR analys* OR outcome* OR useful* OR benefi* OR disadvantag* OR efficacy OR advantag* OR evaluat* OR strength* OR weakness* OR limit* OR learn* OR effect* OR affect* OR esteem OR abilit* OR qualit*) | 7,829,682 |
| 4 | TI=(intervention* OR initiative* OR scheme* OR manage* OR strateg* OR program* OR educat* OR module* OR teach* OR taught* OR course* OR group* OR activit* OR project* OR resource* OR therap* OR support* OR help* OR advice OR class* OR lesson*) | 3,721,265 |
| 3 | AB=(intervention* OR initiative* OR scheme* OR manage* OR strateg* OR program* OR educat* OR module* OR teach* OR taught* OR course* OR group* OR activit* OR project* OR resource* OR therap* OR support* OR help* OR advice OR class* OR lesson*) | 14,123,937 |
| 2 | AB=(child* OR "young pe*" OR "young care*" OR youth OR teen* OR adolescen* OR grandchild* OR grand-child* OR school* OR preschool* OR pre-school* OR paed* OR pedia* OR kid OR kids) | 1,271,200 |
| 1 | TI=(child* OR "young pe*" OR "young care*" OR youth OR teen* OR adolescen* OR grandchild* OR grand-child* OR school* OR preschool* OR pre-school* OR paed* OR pedia* OR kid OR kids) | 929,578 |

APPENDIX 3: Findings Extracted from Each Study

| Alpha Code | First Author (Year) | **Relevant Qualitative Finding with Numeric Code:** *Illustrative Support.* (Credibility Rating) | Relevant Quantitative Findings | **Qualitized Findings with Numeric Code** |
| --- | --- | --- | --- | --- |
| A | Baker (2019) | **1. Children responded positively and empathetically to animations and videos:** *“...when teachers explain something I don’t always understand... but the videos, that made it more interesting and I, like, got everything they were talking about.”*  *“those kids on the video, I really feel for them”.* (Unequivocal)  **2. Activities were received enthusiastically and were a part that were liked best by children:** *Was “fun” (e.g., the brain game or poster) or because it “made you think about it a lot” (e.g., the letter writing)* (Credible)  **3. Learning about the occurrence of younger-onset dementia and the non-contagiousness of the condition stood out to children:** *“when I heard that you could get it [dementia] in your 30s I was really*  *astonished”* (Credible)  **4. Children enjoyed and benefitted from understanding dementia’s impact on different interests and functions through being taught about the brain:** *“it was nice to know about the brain...I just thought it was one big thing...but there was lots of different parts.” “I thought, how do people with dementia do stuff, because they forget everything, but later in the program I found out that only parts of their brain stop working, so they can still remember some things, and how to walk and stuff like that”* (Unequivocal)  **5. Multimedia dementia education resources can improve children’s confidence with supporting people with dementia:** *“I wouldn’t have known what to do, I’d probably get all scared... now I’d help them and I wouldn’t treat them like they’re a little kid, I’d treat them like you’d treat a normal human being.” “I knew he [my grandad] has it and what to do but I didn’t know there was so much you could do that would help him feel better about himself.” “if you didn’t do it, people would probably just walk past someone with dementia”.* (Unequivocal)  **6. Children respond positively to opportunities to teach others about dementia:** *“I, for once, got to teach my parents something ... they only knew the name of it...they knew that it was a memory loss thing, but they didn’t know anything else about it.”* (Credible) | Kids4Dementia participants (n = 136) had improved scores on the validated questionnaire, when compared to the control school participants (n = 67), especially when students had not heard of dementia before (Time x Group x Dementia Familiarity interaction, F(1, 191) = 5.28, *p* = .023, partial *η*^2^ = 0.027). Improvements had a medium effect size of 0.45 for children who had previously not heard of dementia, and a small effect size of 0.22 for children who had previously heard of dementia. | **7. Multimedia dementia education sessions can improve children’s attitudes relating to the personhood of people living with dementia, dementia stigma and understanding of dementia** |
| B | Burns (2020) | **1. Pairing art-based and formal teaching in sessions can help children understand the experience of memory for people living with dementia as involving fading or lost memories, and disorder/chaos:**  *As detected through how children updated a piece of artwork post-intervention to create senses of disorder and chaos.* *Illustrated with art examples on p.174 of the article*. (Credible) 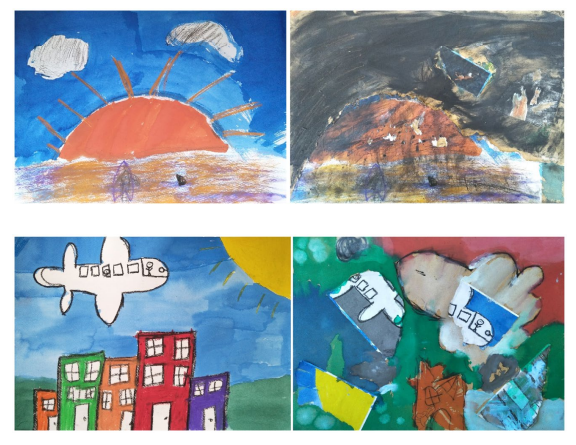 | Paired t-test results were collected for 14 different domains of the Kids Insight into Dementia Survey. Scores in seven showed significant positive improvements when measured with a paired t test:  1. “Spending time with people with dementia can be fun”. (t = 3.867, *p =* .000, r = .208).  2. “People with dementia can feel when others show them love and  understanding”. (t = 2.408, *p =* .019, r = .199).  3. “People with dementia have hobbies and interests”. (t = 2.119, *p =* .038, r = .369).  4. “People with dementia can be creepy”. (t = -.223, *p =* .002, r = .263).  5. “You can ‘catch’ dementia from other people”. (t = -7.721, *p =* .000, r = .231).  6. “Dementia is when something has gone wrong in your brain”. (t = 4.433, *p =* .000, r = .201).  7. “Dementia is unpredictable”. (t = 2.592, *p* = .012, r = .283).  Items with non-significant positive improvements demonstrated a ceiling effect, as initial responses demonstrated initial positive views. A non-significant negative change was found when a paired t-test was conducted for the pre- and post-intervention scores for “I wouldn’t really know what to say or do if I met someone with dementia”  (t = 4.433, *p =* .000, r = .201). | **2. Pairing two art-based and one formal dementia teaching hour-long lessons can improve children’s understanding of people living with dementia.**  **3. Pairing two art-based and one formal dementia teaching hour-long lessons did not improve children’s confidence with interacting with people living with dementia.** |
| C | Chow (2018) | **1. Having quality visits with people living with dementia can help children become more insightful about and understanding of people with dementia:** *“although everyone with dementia is unique, they are just like us – they have different needs, likes, dislikes, and they have ups and downs.” “While they are often forgetful and confused, they can be quite lively at times.” “They’re just like us, and they need all the love and support that they can get”; “Each individual has a distinct life story and should not be simply labeled by their disease.”* (Credible) | Quantitative questionnaire data are not shared by the authors. | Not Possible |
| D | Di Bona (2019) | **1. Completing lifestory books can help children to interact with people living with dementia:** *The authors reported that “some children stated they were useful in guiding their questions”.* (Credible).  **2. Completing lifestory books are not preferable for all children due to some preferring spontaneous listening and chatting or disliking the literary demands:** *The authors reported that some “children [reported] preferring listening and chatting more spontaneously. Two reported “hating” the reading and writing involved in the books”.* (Unequivocal).  **3. Visiting people with dementia can help children to learn about how to support them:** *“nice to visit, to find out what help they needed and to be able to help*  *them” “You should not*  *rush them or get angry but offer to help them as they might have dementia”.* (Credible).  **4. Visiting people with dementia can help children to relate to and understand people living with dementia:** *“people with dementia were like normal people, funny and told jokes” “You should treat people with dementia with kindness”.* (Credible).  **5. Interventions need to provide children with certainty about dementia’s cause:** *uncertainty was detected by the authors as one child asked “Can you catch it? Is it a virus?”.* (Credible). | Quantitative results were not analysed as they combined the scores of children who did and did not receive the intergenerational intervention programme element. | Not Possible |
| E | Farina (2020a) | **1. A single dementia education session can give children new understandings about dementia in terms of its different types, symptoms and experiences of people living with dementia:** *“I thought it was really informative, there was obviously some parts that I think a few people knew in the class but I think the majority of it, because it's not really spoken about a lot and I think it, a lot of people learnt a lot from it, whether it was like, little parts of the illness or, the fact there's lots of different types, how it affects you, because I think most people thought it just affected how you thought, like I especially didn't know that it could really affect your everyday life like trying to do simple tasks like your mobility and stuff’ (female, focus group 4) “it gave me more like awareness of it [dementia], because before like I just thought it's just that you forget things, but like with that class I learned that they do forget things but they remember like old memories and they keep their feelings and everything’ (female, focus group 2) “because my Gran has dementia, I could, because of that, I can like know more things about what she's feeling’ (male, focus group 2) “I didn't know you like you still remember like the emotions so I thought that you just forget like everything’ (female, focus group 4)* (Unequivocal)  **2. A single dementia education session can give children new understandings about dementia in relation to their perceptions of people living with dementia and empowered them to support these people:** *“It made me feel more positively about the people that have it’ (female, focus group 2) “Like before the thing [dementia awareness class], I thought like dementia was like an illness it was just a terrible thing, and now I kind of realized it's not that bad’ (male, focus group 2) “After the session I feel like I'd find it much easier to go see someone with dementia and what it’s like, because I know kind of what I'd be expecting and if I saw them doing certain things I'd know how to deal with it a bit more than before’ (male, focus group 3). Participant: ‘If someone's like living with dementia you still see them as who they are not just what they have, like if someone has dementia you don't just see them as someone suffering from dementia, they're still a family friend, a parent, a carer, like they're still the same person, you may just need to adjust what you say or how you live around them, but they're still the same person’. Interviewer: ‘do you think you had that opinion before or did it change because of the session’. Participant: ‘I had that opinion already but it came a bit more stronger like after’ (female, focus group 3)* (Unequivocal)  **3. A single dementia education session can improve children’s intentions to spend time with and work with people with dementia in the future:** *“I would like to [spend time with people with dementia] as well, like, and then it might, because we might learn about what dementia is and it might help them, it might help us like, understand if they forget something and I'd just like to spend time with them a bit more’ (female, focus group 1) “I'd want more friends to come and see me, so I wouldn't mind spending time with someone with dementia’ (female, focus group 1) “yeah I would work with them [people with dementia] because I know more about it than I did, it's like easier to understand how they're feeling’ (male, focus group 2)* (Unequivocal)  **4. Children benefit from education interventions being delivered positively:** *“The staff [facilitator] are really like nice and trying to engage everyone like with the games, and I think people that wouldn't usually like get involved I think they were trying to listen because she was really genuinely quite a nice person, friendly, and it came across’ (female, focus group 4) “I think everyone should receive the same because they might feel, it might like help people that have people with dementia, it might make them feel like they can open up, but then it could help other people in the class who don't have a family member with dementia, and like, they might not know a lot about it, and then they might be put in another session because people might think they have someone with dementia so they know, or they might not ‘cause [trails off]’ (female, focus group 1)* (Unequivocal)  **5. Children benefit from interventions being interactive:** *“everyone was kind of focused and involved, she kept everyone kind of not just slipping out of concentration everyone was focusing on what she was talking about’ (male, focus group 3)”* (Credible)  **6. Children believed around an hour was appropriate for a dementia education session:**  *“Overall, the length of the session was seen as acceptable and appropriate. The Dementia Friends sessions within this study lasted*  *50 min to 1 h, though ultimately the length of time is dependent on how long it takes to cover the predefined content. Participants*  *believed that the session was long enough to enable them to understand and remember the content of the session but was not too*  *long that people lost their focus or concentration”* (Credible)  **7. Children appreciate interventions providing in-depth factual detail about dementia, including its different types, causes and symptoms:** *“I think possibly like, if they could go more in depth on how it happens, and not just the … effects, but more about the causes’ (male, focus group 2) “because she [EJ] was saying about some of the signs of it, like the early signs of it like losing your short term memory and stuff and I think it's good for you to, because then you can look out for your family members, try kind of like sense how they're going’ (female, focus group 4)* (Unequivocal)  **8. Children appreciate interventions that involve learning from people with dementia:** *“I think that [being taught by someone with dementia] would be quite an emotional way for it to be brought across, I think that would really affect the students more than just someone who doesn't have it, but obviously that might be more difficult for the person to do it than someone else just coming up and telling them about it’ (female, focus group 4) “It [being taught by someone with dementia] would probably reach more people than if it was a teacher’ (male, focus group 4)* (Unequivocal)  **9. Children appreciate interventions providing practical ways of helping, identifying and engaging with people living with dementia:**  *“what you could do just as one person, um like actually do, obviously we know um part of dementia friends is like an awareness but like, that's just almost an awareness, what actions you could take if you had a family member with dementia’ (female, focus group 4) “actively how you could help that person and or other people in your community maybe anywhere local who does like, because obviously you have places like the hospice, or like nursing homes, is there anywhere like around us a lot of people with dementia can get help from that we can like support’ (female, focus group 4).* (Unequivocal)  **10. Children believe it would be useful for dementia education interventions for young people to be freely accessible:** *“I agree that everyone should have the same lessons because even if you don't have a family with dementia now then you can't say the same for in the future when you haven't had the same lessons so you can learn all about it’ (female, focus group 1) “later on they may have a family member who has dementia and then not having that lesson may really like make them stuck or whatever’ (female, focus group 1).* (Unequivocal)  **11. Children desired more than one dementia education session:**  *“Personally, I think it would be good to have a refresher session because they can tell you about how people get it or like, what goes on like in their mind and loads of other stuff about it as well’ (male, focus group 2) “maybe if we had like two more sessions in it or something and like, maybe one could be like with a person with dementia and the other could be like how to like help people’ (female, focus group 1).* (Unequivocal) | Not Applicable. | Not Applicable. |
| F | Farina (2020b) | Not applicable | No statistically significant score change differences were found between the intervention and control group were found in either the dementia awareness (t=0.03, *p=*0.98, d=-0.003) or insights (t=-1.02, *p=*0.31, d=0.14) questionnaires. Within the multivariate models, after factoring covariates, there was no significant main effect of Time (F (1,278) = 0.58, p = 0.45, *η*^2^ = 0.002) or Time x Group (F (1,  278) = 0.001, p = 0.98, *η*^2^ < 0.0001) for the insights questionnaire. With the attitudes questionnaire there was no significant main effect of Time (F (1, 238) = 1.23, p = 0.27, *η*^2^ = 0.005), or Time x Group effect (F (1,238) = 1.44, p =0.23, *η*^2^ = 0.006). No significant difference of change scores was found between the groups regarding their willingness to work with people with dementia in the future (Z = -1.29, *p* = .20, d = .17, 95% CI (.04–.30)). Descriptive quantitative data revealed that the session was enjoyable and subjectively improved participants attitudes and knowledge. | **1. A single dementia education session does not objectively, but does subjectively, improve understanding of people living with dementia.**  **2. Children enjoy single dementia education sessions.**  **3. A single dementia education session does not improve behavioural intentions to support individuals living with dementia by working professionally with them.** |
| G | Gibson (2018) | **1. Interactive sessions can help children to learn that they can support people with dementia through socially interacting with them, offering assistance and communicating appropriately with them:**  *“Spend time with them’ “Chat to them to make them feel less lonely’ “Take them for a walk’ “Help them get off the bus and tell them when their stop is’ “If they are confused try and help them make their decision’ “Speak slowly and quietly’ “Be polite’ “Don’t get annoyed with people who have dementia’* (Unequivocal) | Not Applicable. | Not Applicable. |
| H | Liao (2022) | Not Applicable. | After controlling for participation in volunteer services for older people, age, school grade and gender, the interaction effect of group by time in the 8-week exergaming group showed that there was a significantly improved attitude toward dementia at post-test, and at the 1-, 3-, and 6-month follow-ups (95% confidence interval (CI) 2.585 to 10.908, *p* = .001; 95% CI 3.559 to 17.365, *p* = .003; 95% CI 5.000 to 18.612, *p* = .001; 95% CI 5.121 to 18.210, *p* < .001, respectively). The attitude toward dementia in the 8-week companion group was also significantly better than that of the control group both at the post-test and at the 6-month follow-up (95% CI 1.693 to 12.074, *p* = .009; 95% CI .263 to 13.023, *p* = .041, respectively). For the 5-week exergaming group, the attitude toward dementia showed no statistically significant difference compared with the control group at post-test, and at the 1-, 3-, and 6-month follow-ups. However, the attitude toward dementia in the 5-week companion group was significantly better than that in the control group only at post-test (95% CI 0.416 to 9.657, *p* = .033). | **1.** **Intergenerational interactions with people living with dementia, via either playing physical exercise-utilising video games or accompanying them during their daily activities, can significantly improve adolescents’ social comfort and knowledge about dementia**  **2. Weekly intergenerational interventions need to run for more than 5 weeks for meaningful changes in knowledge of and social comfort towards dementia to be detected.** |
| I | Mastel-Smith (2022) | **1. Children feel interacting with people living with dementia can help them to understand people with the condition:** *“Meeting people with dementia “helped me understand . . . more about what people experience every day” and was more effective than “reading about it.”* (Unequivocal)  **2.** **Online learning management systems for hosting dementia education content are helpful:** *“[system was a] great resource” and “easy to navigate”.* (Credible)  **3.** **Children enjoy learning about dementia from activities:** “*“My favourite one was where we learned about the modifiable versus non-modifiable risks factors.’ Students suggested improving the session by adding more activities.* (Credible)  **4.** **Virtual dementia simulations can help children to understand dementia:** *“helped us to see what living with dementia feels like on a daily basis, and it was a good way to put us in their shoes.”* (Credible)  **5.** **Virtual teaching from and meeting healthcare professionals, dementia patients and dementia carers can help children to feel confident with supporting a person living with dementia:** *students were “pretty confident . . . that I would be able to communicate with someone with dementia.” “It’s important to be understanding and patient, don’t treat them like a kid, like a baby because they’re not. They’re still a person with feelings,” “they need a bit of time to process what you said”, I’ve “learned to adapt or rearrange my words”, and, “the level of vocabulary I will use for a person living with dementia, it would depend on how the disease has progressed, how much it has progressed because depending on that, you can rephrase the words if they don’t understand it or try to find some way in which to say things that they would be able to understand.”* (Unequivocal)  **6.** **Virtual teaching from and meeting healthcare professionals, dementia patients and dementia carers can help children understand and empathise with the personhood of a person living with dementia:**  *“People can overcome some of its more intense symptoms and they can live a normal and happy life for as long as they can.” “Everyone just thinks that their life is just over and they can’t do anything any more, but talking to so many people with dementia, you see how many things you can still do, and it doesn’t necessarily mean that you just sit on the couch all day and just do nothing.”*  (Unequivocal)  **7.** **Students felt it was important to learn about the disease and brain elements of dementia:** “*There’s a lot to understand about dementia that most people don’t know’ “I really learned just so much – that there’s just so many different aspects of the disease that could be delved into and that it’s just important to have a basic understanding of it.” More information about how dementia affects the brain was recommended* (Unequivocal)  **8.** **Children benefit from learning about how emotions in dementia can be separate to memories:**  *“Someone with dementia, they might not remember you, but they know that you’re important to them or that they love you.”* (Credible)  **9.** **Children enjoy opportunities to interact with peers when learning about dementia:** *“Maybe we could do things more interactional with each other as students . . . it’s cool to talk to other people who are learning at the same time as you to see how they’re feeling.”* (Credible)  **10.** **Children prefer to be allocated a longer time period for sessions of around 2 hours which could be terminated early when learning about dementia** *Students agreed that extending sessions to two hours was desirable so that, “Maybe just already setting it at two hours would be better because it’s better to finish early than have too long of a meeting . . . And you don’t have to worry about rushing.”* (Credible)  **11.** **Children can dislike having too much independent work and reading and prefer learning through presentations**  *Perceptions of the pre-work varied. It was felt by one student that for some modules the pre-work was too long. Presentations both reiterated what was in the pre-work and provided more in-depth information which “helped (her) understand better.”* (Credible) | Not applicable | Not applicable |
| J | Masuda (2019) | **1. Technology malfunctioning can affect children’s experiences when learning about dementia:**  *"the talk stopped suddenly, and it was hard to hear", "it was a one word"* (Credible) | Significant improvements for quiz scores were found for 3 questions: "Dementia can Affect anyone" (*p* <0.001), "Dementia patient and trouble family" (*p* = 0.002), “I know about elderly support networks" (*p* = 0.004). Non-significant improvements were found for “everyone is easy to live in the town that will help people in need (*p =* 0.102) and “if there is a person who is in trouble, I want to help” (*p =* 0.276), perhaps due to a ceiling effect where initial positive responses were high. | **2. Learning about dementia through a humanoid robot simulating and teaching about dementia can help children to understand and improve their knowledge about dementia.** |
| K | Nazir (2015) | **1. Children find brain models helpful for understanding dementia:** *“The brain model helped me to understand how dementia affects the mind.”* (Credible)  **2.** **Roleplay can be an enjoyable way for children to learn about dementia:** *“I liked taking part in the role play and pretending to be someone who suffers from dementia.”* (Credible)  **3.** **Activity-based teaching sessions can help children to understand dementia and the care role.** “I think I now will be able to spot signs of dementia in my family members.”, “I used to think that people having dementia were a bit scary. Now I realize that they should be treated like normal people.”, “Thanks for making dementia easy to understand.”,” I had no idea how hard it must be to be a carer.”, “I didn’t know that people with dementia and their carers go through so  much. It’s good that they can get lots of help.”, “What I’ve learnt will help me to become a good carer when I grow up.”, (Credible)  **4.** **Children reflect on opportunities to teach others following dementia education sessions:** *“I told my family what I had learnt. Now they know about dementia.”* (Credible)  **5.** **Children wish to understand the research evidence base around dementia from interventions:** *“It would have been good to learn about research being done for dementia.”* (Credible)  **6.** **Children consider practising diagnosing patients with dementia using standardised examinations** *“I would like to try the SMMSE on anyone who might be having signs of dementia.”* (Credible)  **7.** **Children find case studies of celebrities living with dementia interesting***: “Learning about famous people with dementia would have been interesting.”* (Credible) | 135/150 students agreed to have found the project helpful. | **8.** **Children find activity-based teaching sessions helpful for learning about dementia.** |
| L | Noble (2015) | Not Applicable. | AD symptom recognition improved significantly between the pre- and immediate post-tests for all comparisons (p ≤ .01 for all, χ2 (1) ranged from 6.5 to 91.5.).  Symptom knowledge was better retained 3 months later when the symptoms had also been included in a mnemonic memory aid and song. For included symptoms, all were significantly retained (p < .001 for all comparisons, χ2(1) ranged from 14.3 to 66.0). AD symptoms not in the mnemonic were least retained: paying bills, 35 (59.3%) correct in delayed post-test versus 56 (52.2%) in pretest, χ2(1) = 0.64, p = .42, and misuse of appliances, 47 (73%) versus 35 (52%), χ2(1) = 6.8, p = .01.  Knowledge about dementia neuroscience improved following the intervention. For brain localisation pre- vs post-test comparisons, p = .02 for immediately after and p = .09 for 3 months after; For remembering localisation pre vs post-test comparisons, p < .001 for both immediately and 3 months after. For hippocampus pre vs post-test comparisons, p < .001 for both immediately and 3 months after. | **1. Creative arts-based interventions can improve children’s recognition and neuroscientific understanding of Alzheimer’s disease symptoms**  **2. Children retain information about Alzheimer’s symptoms for longer when it is also included in a memory aid like a mnemonic or song** |
| M | Parveen (2015) | **1.** **Children enjoy learning about types of dementia:** *learning about types of dementia was highlighted by the children as a “best bit” of the intervention.* (Credible)  **2.** **Children enjoy group discussions of dementia:**  *group discussions about dementia were highlighted by the children as a “best bit” of the intervention.* (Credible)  **3.** **Children enjoy discovering practical ways they can help someone with dementia through joining dementia championing schemes:**  *discovering how to become a dementia friend was highlighted by the children as a “best bit” of the intervention.* (Credible)  **4.** **Children like interactive and physical activities when learning about dementia:**  *The majority of children reported wanting to have more interactive and physical activities.* (Credible)  **5.** **Children like to meet people living with dementia face to face to learn about the experience of living with dementia:**  *One student suggested that they would have liked a person living with dementia to attend the session in order to provide a face-to-face account of their experiences.* (Credible)  **6.** **Children enjoy videos when learning about dementia:**  *videos were highlighted by the children as a “best bit” of the intervention.* (Credible) | Perceived knowledge improved from 4.5 to 8 on average.  On average, the information was rated as 7.8 for being understandable.  Enjoyment of the session received an average score of 7.9.  36/38 students would recommend the session to a friend and others. | **7.** **A single-session dementia awareness programme is enjoyable, understandable and can improve children’s perceived knowledge of dementia.** |
| N | Pawlicka  (2023) | **1. Pairing art-based and formal teaching in sessions can help children understand the challenges, memory symptoms, and frustrations related to dementia:**  *“It made me a bit sad. Just that kids have to live with someone or know someone in their family that doesn't understand...like they could come up to cuddle them and, “Oh, I'm so happy to see you”, but then, “Who are you?”, so it could be quite heartbreaking [...]that they don't know who you are and you don't feel like you're loved by your grandparents or parents or whoever it is that you're related to. Yeah, so that was sad*.” *“The person has forgotten and is really trying to remember, but they’re just trying to put the pieces together.” “At the start I didn't really know what dementia was,[...] so after this, I felt like very sad for the people that have, like, people in the family that have dementia and they forget their...and it just feels very sad.”*  (Unequivocal)  **2. Pairing art-based and formal teaching in sessions can help children develop resilience and positive coping strategies related to dementia.**  *“Learning more is like what I want to know...and you get used to it just in case...like maybe your family member gets dementia and then you can go ‘Okay. I know what's going to happen’. I shouldn’t get mad at them because it's not their fault, it's...they’re just get-ting this dementia.” “I could remind her, her name. I would remind her, her family and I'd remind her that it's...it's not that bad when you have love.”* (Unequivocal)  **3. Children find art a useful and enjoyable medium for expressing their thoughts, feelings and learning about dementia.**  “I think it was really fun...I liked that we got to do art because in most of the things we do at school there isn't really much art in it.” “I thought it was really fun. I wish we could do it again...you got to put your feelings and everything on the page so you could express and not keep holding it in until you’re, like, who knows, seventy-six!.” “It was fun...The artwork, and, like, getting to see other people's artwork and how they felt in all those memories.” Students discussed how they were able to manipulate artwork to express their understanding of dementia. (Credible) | Not Applicable. | Not Applicable. |
| O | Ritchie (2023) | **1. Visual models can help children to understand the neuroscience of dementia**: *“I liked when we were having the model of the brain because you can understand about what dementia is it, it was quite interesting to look at what parts of the brain get affected when people have dementia”* (Unequivocal).  **2.** **Sharing examples of assistive technology can help children to understand and adapt support strategies for people living with dementia:** *“You see the phone one with the images, yeah. I think it will help the people with dementia because if they forget a number they can just press the button with the picture.’ “The numbers were really big because with the goggles that we wore in the third group that we were in, the eyesight was quite bad and it was quite hard to see so with the big numbers it was a lot easier to see for people with dementia’* “*My favourite bit was the voice-recorder you could record your voice and then say someone with dementia always went out at night time thinking it was day so you could put the message on that recorder said "You can’t go outside, Mum, it’s night-time", so it’s kind of to remind them…..I kind of disliked how*  *instead of- I think it should have when it detects movement, instead of you having to push a button, because what if the person forgets to push the button?’.* (Unequivocal)  **3.** **Including information about dementia risk factors can create worry for some children regarding their own dementia risk: *“****Maybe it’s good a wee bit...but if it scares some person because they get dementia sometimes of people but they don’t get better but if people are scared to get dementia afterwards’.* (Credible)  **4.** **Interventions including a mixture of diverse interactive activities can help children to learn how to support a relative living with dementia:** “I learned a lot of new things and how I can help my Grandad if I was ever around him” (Credible)  **5.** **Children reflect on opportunities to educate their parents about their dementia education:** “*I told my Mum and my Dad and he said it was really good so you can understand more about what’s going on with my granddad and everything.*” (Credible)  **6.** **Simulation and sensory experiences, particularly with scent boxes and goggles modified to reflect vision in dementia, can help children to understand and discover how to alleviate the non-memory related symptoms of dementia:** *“because I only thought that they forget things but then it effects their senses and everything.’ “They might see it differently as us, we see it quite normally and they see it quite hard because they don’t know, they don’t know what they’re doing anymore because of their dementias’ “if they’re going to the shop and they’re like’ ooh, I don’t know what to get’ they like smell it and like’ that smells like that, so I could get that’. “I felt quite empathetic because I was like imagining what it would be like to like, live with a person with dementia.’ (Bobby, FG1)* (Unequivocal) | Not Applicable. | Not Applicable. |
| P | Sakai (2014) | Not applicable. | Reading and discussion of storybooks improved child Alzheimer’s disease-related knowledge (F(2, 108) = 165.525, *p* < .001), attitudes (F(2, 108) =19.395, *p* < .001), and willingness (F(2, 108) = 12.169, *p* < .001). It did not influence positive (F(2, 108) = 1.512, *p* = .227) or negative affect (F(2, 108) = 2.203, *p* = .119). Knowledge  significantly increased after reading the book (*t*(54) = -12.873, *p* < .001) but additional increases were not found after the discussion (*t*(54) = -1.687, *p* = .097). Children had significantly more positive attitudes about Alzheimer’s disease after reading the book (*t*(54)= -4.607, *p* < .001, (baseline mean = 27.33, after reading = 30.05)). Attitudes about Alzheimer’s disease did not become more positive after having the discussion  (*t(*54) = -.728, *p* = .470 (after discussion, mean = 30.40)).  Even though the book distinguished between Alzheimer’s disease and senility, 40% of the children responded incorrectly to the survey item related to this knowledge at baseline, 47% after book reading and 44% after the discussion. Items requiring inference were also misunderstood.  Children reported similarly high levels of subjective Alzheimer’s disease knowledge gained after reading the book, with 44 (80%) stating they learned “quite a bit” or “a lot” after reading the storybook.  Fewer children reported feeling uncomfortable with (53% before versus 33% after reading the book) or scared of (35% versus 20%) people with people with Alzheimer’s disease after reading the book.  Children were also less likely to believe that people with Alzheimer’s disease are unable to do things that they want to do (44% before versus 64% after).  Children also demonstrated more positive attitudes about people with Alzheimer’s disease after reading the storybook, with an increased number reporting that it could be fun to spend time with people with Alzheimer’s disease  (53% before versus 84% after reading the book). Children’s attitudes did not change on all items (e.g., an item related to if someone with Alzheimer’s disease needed help with something I would help them).  Before the intervention, the average children’s willingness to interact with someone with Alzheimer’s disease score was 26.27  (SD = 5.15), with mean scores after reading and discussing the storybook of 28.33 (SD = 5.52) and 28.44 (SD = 5.71), respectively. Scores after reading (*t*(54) = -3.731, *p* < .001) and discussing (t(54) = -3.760, p < .001) the storybook were both significantly higher than pre-intervention, but not significantly different from each other (*t*(54) = -.348, *p* = .729).  More children wanted to spend more time with people with Alzheimer’s disease after the intervention (quite a bit/a lot: 51% versus 77%). The percentage of children reporting they would want to give someone with Alzheimer’s disease hugs and kisses “a lot” increased from 22% to 38%. The percentage of children who did not want to give hugs and kisses “at all” did not change (20% before reading versus 22% after reading).  Children did not demonstrate greater willingness to do more chores around the house after the intervention, as nearly identical numbers of children were keen to do this activity pre- and post-intervention.  All but one child reported liking the book at least “a little bit”. Almost half of the children indicated that they liked reading the book “a lot” (n = 27, 49%). Many children reported that they were “quite likely” (n = 14, 26%) or “very much likely” (n = 20, 36%) to read the book again. Many children recommend the book to others at roughly the same rate, with most children saying they were “quite likely”  (n = 20, 36%) or “very much likely” (n = 20, 36%) to recommend the book.  Girls were more likely to report interest in reading the book again (r = .330, *p* =.014) and recommending the book to a friend  (r = .354, *p* = .008).  Children varied in the extent to which they liked the discussion element of the intervention. Five children (9%) indicated that they did not like the discussion at all, 13 (24%) liked the discussion “a little bit,” 8 (15%) “quite a bit,” and the remaining 29 (53%) “a lot.”  Higher parent education correlated with more negative child attitudes about Alzheimer’s disease (*r =* -.349, *p =* .009) and greater negative affect (*r =* .285, *p =* .035) after the discussion. Parent work experience was negatively associated with children’s attitudes after reading (*r* = -.352, *p* = .008) and discussing the book (*r* = -.349, *p* = .009) and willingness to interact with people with Alzheimer’s disease after reading the story book  (*r* = -.289, *p* = .032). | **1. Reading an Alzheimer’s disease-related storybook can improve attitudes and objective and self-reported knowledge about the condition.**  **2. Reading and discussing Alzheimer’s disease related storybooks does not seem to affect the distress levels of children**  **3. Reading and discussing storybooks related to Alzheimer’s disease does not clear up misunderstandings of all dementia facts, particularly when not explicitly shared in the book.**  **4. The impact reading and discussing storybooks related to Alzheimer’s disease had on children’s willingness to support individuals with Alzheimer’s disease was variable and stronger for behaviours related to spending time with people with the condition than those related to physically supporting them.**  **5. Most children like reading storybooks related to Alzheimer’s disease.**  **6. Not all children enjoy formally discussing Alzheimer’s disease-related storybooks when asked questions about it by a parent.**  **7. Where parents lead storybook reading and discussion interventions, the parent having higher education and previous dementia work experiences can be negatively associated with children’s attitudes and willingness to engage with people with Alzheimer’s disease post-intervention.** |
| Q | Smith (2020) | Qualitative results were not analysed as they combined the feedback of children who did and did not receive the intergenerational intervention programme element. | Post-hoc pairwise comparisons revealed improvement in dementia knowledge and attitudes from baseline to post-programme (*p* = .001) and baseline to six-month follow-up (*p* < .001). No difference was found between the post-programme results and the 6-month follow-up (*p* = .21). There was a main effect of dementia knowledge at baseline (F[1, 56]= 5.79, *p* = .020, partial η2= .094) and a time x dementia knowledge interaction (F[1.8, 100.9] = 3.24, *p* = .048, partial η2 = .06). For each questionnaire factor, there was a main effect of time: personhood (F[2, 112] = 28.32, *p* < .001, partial η2 = .34), stigma (F[1.7, 95.86] = 29.07, *p* < .001, partial η2 = .34) and knowledge (F[2, 108] = 22.08, *p* < .001, partial η2 = .29). For both stigma and knowledge, post-hoc pairwise comparisons revealed this was due to significant improvement from pre- to post-programme (both *p* < 0.001), and pre-programme to 6-month follow-up (both *p* < 0.001), but not from post-programme to six-months follow-up (both *p* = 1.00). For personhood, scores increased from pre- to post-programme (*p* < 0.001) and long-term follow-up (*p* < 0.001) and also from post-programme to 6-month follow-up (*p* = 0.007). There were no other main effects or interactions for the individual factors. There was no augmentation effect related to whether children were in the excursion or non-excursion condition. Results from the follow-up survey indicated there were no decline in scores from post-programme to 6-month follow-up (*p* = 0.21). | **1. School-based dementia teaching over the course of two months with or without an intergenerational experience can result in prolonged objective improvements in children’s dementia attitudes and knowledge.**  **2. Intergenerational experiences do not augment the objective improvements to children’s dementia attitudes and knowledge obtained by receiving just school-based dementia teaching over the course of 2 months.** |

APPENDIX 4: Studies Excluded After Full Texts Were Sought for Retrieval

| **Record Citation** | **Reason for Exclusion** | **Elaboration** |
| --- | --- | --- |
| **Records not retrieved as full text not available (n= 3)** | | |
| Jarrott (2013) | Does not meet accessibility eligibility criteria. | The full text was not retrievable through the university library database, or general Google Scholar searches. |
| Felten and Hyland (2018) | Does not meet accessibility eligibility criteria. | The reference is a poster presentation, for which the abstract is only available on the university library database. |
| Wallace and Horner (2014) | Does not meet accessibility eligibility criteria. | The full text was not retrievable through the university library database, or general Google Scholar searches. The complete study of the pilot is included in the final set. |
| **Outcome measures of interest not provided (n= 8)** | | |
| Bormans and Zwakhalen (2022a) | Does not meet outcomes of interest eligibility criteria. | Outcome measures are not collected and analysed; future study protocol described. |
| Foy *et al.* (2023) | Does not meet outcomes of interest eligibility criteria. | Outcome measures are mostly collected from adults, and the outcome measures collected from children shared are not explicitly about their dementia learning. |
| McGarry *et al.* (2021) | Does not meet outcomes of interest eligibility criteria. | Intervention is described and not evaluated for its effectiveness in children. |
| ‘Cross-curricular dementia awareness toolkit developed for use in schools’ (2013) | Does not meet outcomes of interest eligibility criteria. | Intervention is described and not evaluated for its effectiveness in children. |
| Watson and Smith (2020) | Does not meet outcomes of interest eligibility criteria. | Intervention is described and not evaluated for its effectiveness in children. |
| Rylance and Pendleton (2015) | Does not meet outcomes of interest eligibility criteria. | General work conducted to explore different variations of an intervention is described. |
| Pearce (2016) | Does not meet outcomes of interest eligibility criteria. | Intervention is described and not evaluated for its effectiveness in children. |
| Bormans and Zwakhalen (2022b) | Does not meet outcomes of interest eligibility criteria. | It measures the usefulness of drawings as an outcome measure for interventions instead of measuring the effectiveness of the intervention itself. |
| **Exposure eligibility criteria not met (n= 1)** | | |
| Atkinson and Bray (2013) | Does not meet exposure eligibility criteria due to aggregating different intervention programmes. | It is unfeasible to validly extract the results of the 13 separate intervention studies from the report due to its intended synthesised nature. |
| **Outcome measures not collected from children (n= 5)** | | |
| Murashima *et al.* (2018) | Does not meet outcomes of interest eligibility criteria. | Outcome measures were collected from five adult experts in community welfare. |
| Liu, Tuah and Miao (2022) | Does not meet outcomes of interest eligibility criteria. | Outcome measures were collected from six adult experts in user experiences, serious games and persuasive computing. |
| Skropeta, Colvin and Sladen (2014) | Does not meet outcomes of interest eligibility criteria. | Outcome measures collected from child carers. |
| Hull *et al.* (2019) | Does not meet outcomes of interest eligibility criteria. | Outcome measures are provided from researcher and teacher perspectives. |
| Whitehouse, Kruger and Whitehouse (2018) | Does not meet outcomes of interest eligibility criteria. | The outcome measures provided are from the perspectives of the authors. |
| **Record discussed ideas for relevant interventions instead of testing delivered interventions (n= 5)** | | |
| Browne *et al.* (2022) | Does not meet outcomes of interest eligibility criteria. | Discusses children’s ideas about what would be useful to include in a dementia storybook intervention. Evaluation data about the book from children are not provided. |
| Masterson-Algar *et al.* (2023) | Does not meet outcomes of interest eligibility criteria | Details the co-design of an e-Health dementia intervention with children, providing their insights on what would be helpful for this kind of intervention. Does not deliver the intervention to children. |
| Parveen *et al.* (2020) | Does not meet outcomes of interest eligibility criteria | Focus group discussions with children about what they would theoretically like to be included in dementia education interventions. |
| Baker *et al.* (2018) | Does not meet outcomes of interest eligibility criteria | Focus group and interview discussions with children about what they would theoretically like to be included in dementia education interventions. |
| Nichols *et al.* (2013) | Does not meet outcomes of interest eligibility criteria | Focus group discussions with child dementia carers about their needs, which informed a dementia website which is yet to be evaluated. |
| **Incorrect participant age range (n= 8)** | | |
| Tirado-Rafferty *et al.* (2023) | Does not meet population eligibility criteria | Aged 18 and over |
| McNaney *et al.* (2017) | Does not meet population eligibility criteria | Aged 16 to 24 |
| Roberts and Noble (2015) | Does not meet population eligibility criteria | University students aged 21 to 29 years |
| Cheung *et al.* (2022) | Does not meet population eligibility criteria | Aged 18 and over |
| Millenaar *et al.* (2014) | Does not meet population eligibility criteria | Aged 15 to 27 |
| Rathnayake *et al.* (2019) | Does not meet population eligibility criteria | Aged 18 and over |
| Korukcu, Kukulu and Tufan (2018) | Does not meet population eligibility criteria | Aged 22-24 years |
| Bo-Kyoung Song, Ha-Na Kim, and Sang-Hwa Lee (2019) | Does not meet population eligibility criteria | The intervention was not evaluated for a child-centric population as 17-19-year-olds were studied, with over 50% of the sample being 18-19 years old. |
| **The record uses a duplicate data set to a study in the included set (n= 2)** | | |
| Ross (2020) | Excluded to minimise bias resulting from repetition of the same data set. | A report about the journal article by Smith et al. (2020) |
| Masuda, Murashima and Majima (2018) | Excluded to minimise bias resulting from repetition of the same data set. | A less detailed version of the included study by Masuda et al. (2019), which was written by three authors, all of whom also wrote the included study |

APPENDIX 5: Methodological Quality and Level of Evidence Appraisals

**Key:** C#: Tool criterion/question number; RCT: Randomised Control Trial; N: No (failure to meet criterion – indication of lower quality); U: Unclear (unclear if criterion has been met); Y: Yes (criterion met – indication of higher quality).

*Overview of Methodological Quality and Level of Evidence Appraisals*

| First Author (Year) | Study Type | Main Quality Appraisal Tool (*and tools used to inform the main tool’s appraisal)* | Level of Evidence Tool(s) | Level of Evidence Rating(s) | Overall Level of Evidence |
| --- | --- | --- | --- | --- | --- |
| Baker (2019) | Mixed Methods | MMAT for Mixed Methods Studies *(MMAT for Quantitative Non-Randomised Studies and JBI Checklist for Qualitative Research)* | GRADE  ConQual | Low  Low | Low |
| Burns (2020) | Mixed Methods | MMAT for Mixed Methods Studies *(MMAT for Quantitative Non-Randomised Studies and JBI Checklist for Qualitative Research)* | GRADE  ConQual | Very Low  Very Low | Very Low |
| Chow (2018) | Mixed Methods | MMAT for Mixed Methods Studies *(MMAT for Quantitative Non-Randomised Studies and JBI Checklist for Qualitative Research)* | GRADE  ConQual | Very Low  Very Low | Very Low |
| Di Bona (2019) | Mixed Methods | MMAT for Mixed Methods Studies *(MMAT for Quantitative Non-Randomised Studies and JBI Checklist for Qualitative Research)* | GRADE  ConQual | Very Low  Low | Very Low |
| Farina (2020a) | Qualitative | JBI Checklist for Qualitative Research | ConQual | Moderate | Moderate |
| Farina (2020b) | Quantitative | MMAT for Quantitative Non-Randomised Studies | GRADE | Low | Low |
| Gibson (2018) | Qualitative | JBI Checklist for Qualitative Research | ConQual | Moderate | Moderate |
| Liao (2022) | Quantitative | MMAT for Quantitative Non-Randomised Studies | GRADE | Low | Low |
| Mastel-Smith (2022) | Qualitative | JBI Checklist for Qualitative Research | ConQual | Low | Low |
| Masuda (2019) | Mixed Methods | MMAT for Mixed Methods Studies *(MMAT for Quantitative Non-Randomised Studies and JBI Checklist for Qualitative Research)* | GRADE  ConQual | Very Low  Very Low | Very Low |
| Nazir (2015) | Mixed Methods | MMAT for Mixed Methods Studies *(MMAT for Quantitative Descriptive Studies and JBI Checklist for Qualitative Research)* | GRADE  ConQual | Very Low  Very Low | Very Low |
| Noble (2015) | Quantitative | MMAT for Quantitative Non-Randomised Studies | GRADE | Very Low | Very Low |
| Parveen (2015) | Mixed Methods | MMAT for Mixed Methods Studies *(MMAT for Quantitative Descriptive Studies and JBI Checklist for Qualitative Research)* | GRADE  ConQual | Very Low  Very Low | Very Low |
| Pawlicka (2023) | Qualitative | JBI Checklist for Qualitative Research | ConQual |  |  |
| Ritchie (2023) | Qualitative | JBI Checklist for Qualitative Research | ConQual | Low | Low |
| Sakai (2014) | Quantitative | MMAT for Quantitative Non-Randomised Studies | GRADE | Very Low | Very Low |
| Smith (2020) | Mixed Methods | MMAT for Mixed Methods Studies *(MMAT for Quantitative Non-Randomised Studies and JBI Checklist for Qualitative Research)* | GRADE ConQual | Very Low  Very Low | Very Low |

Results of Appraisals with the Mixed Methods Appraisal Tool (MMAT) for Quantitative Non-Randomised Studies (Hong et al., 2018)

| **First Author (Year)** | **Decisions and Justifications for Each Criterion of the MMAT for Quantitative Non-Randomised Studies** | | | | | | |
| --- | --- | --- | --- | --- | --- | --- | --- |
|  | **C1. Representativeness of Sample** | **C2. Outcome and Intervention Measurement Appropriateness** | **C3. Outcome Data Completeness** | **C4. Confounding Variable Control in Design and Analysis** | **C5. Intervention Administered as Planned** | **C6. Research Question Clarity** | **C7.**  **Collected Data’s Relevance for Question** |
| Baker (2019) | Y | Y | Y | Y | N | Y | Y |
| *Justification* | *195 children selected across a range of schools with data about their varied sample characteristics shared.* | *Use of a recognised dementia insight survey to collect quantitative data about the intervention’s ability to improve dementia understanding.* | *Full survey data is available along with statistics and effect sizes.* | *Confounding variable (prior dementia familiarity) was controlled for.* | *Fourth school was not recruited as planned, resulting in differences in group sizes (although this wasn’t thought to significantly impact the ability to detect an intervention effect).* | *Question is stated clearly at the end of the introduction.* | *Collected data is relevant to answer the question about the intervention’s effectiveness.* |
| Burns (2020) | Y | Y | N | N | N | Y | Y |
| *Justification* | *Consent gained for and study conducted on 117 children out of a possible 131 (89%) in the school sampling pool, which covered a range of school classes and ages.* | *Use of a recognised dementia insight survey to collect quantitative data about the intervention’s ability to improve dementia understanding.* | *Full survey outcomes were only shared for those where significant improvements were found.* | *A confounding variable was teacher prioritisation of the survey filling task, resulting in missing data for some students.* | *Teachers did not distribute the surveys equivalently as planned.* | *Research question is clearly stated at the end of the introduction.* | *Collected data is relevant to answer the question about the intervention’s effect on dementia understanding changes.* |
| **First Author (Year)** | **C1. Representativeness of Sample** | **C2. Outcome and Intervention Measurement Appropriateness** | **C3. Outcome Data Completeness** | **C4. Confounding Variable Control in Design and Analysis** | **C5. Intervention Administered as Planned** | **C6. Research Question Clarity** | **C7.**  **Collected Data’s Relevance for Question** |
| Chow (2018) | N | Y | N | U | Y | Y | Y |
| *Justification* | *Sample included only four volunteer high school students selected, meaning children who may be less eager to learn about dementia are not represented. Also, a very small sample size.* | *Use of relevant surveys to collect quantitative data about the intervention’s ability to improve dementia understanding.* | *Full survey outcomes for each question and student were not shared.* | *It is not clear how potential confounding variables were controlled for.* | *The intervention was delivered as planned.* | *Research aim is clearly stated at the end of the introduction.* | *Collected data is relevant to answer the question about the intervention’s impact on knowledge and attitudes.* |
| Di Bona (2019) | N | Y | U | N | U | Y | Y |
| *Justification* | *Children were specially selected to be involved with the study.* | *Use of relevant surveys to collect quantitative data about the intervention’s ability to improve dementia understanding.* | *Pre and post-curriculum scores are provided for each survey question, but in the discussion, it is revealed that questions were left unanswered, so it is unclear how many students the questionnaire data is provided for.* | *The questionnaire was deemed to not validly measure the dementia teaching provided.* | *Unexpected problems arose with the questionnaire suitability and there was debate about how well the teachers had delivered the programme as intended.* | *Research aims are clearly stated at the end of the introduction.* | *Collected data is relevant to answer the question about the intervention’s effect on dementia knowledge and awareness.* |
| **First Author (Year)** | **C1. Representativeness of Sample** | **C2. Outcome and Intervention Measurement Appropriateness** | **C3. Outcome Data Completeness** | **C4. Confounding Variable Control in Design and Analysis** | **C5. Intervention Administered as Planned** | **C6. Research Question Clarity** | **C7.**  **Collected Data’s Relevance for Question** |
| Farina (2020b) | Y | Y | Y | Y | Y | Y | Y |
| *Justification* | *Large sample size of 301 teenagers with data about their varied sample characteristics shared.* | *Use of relevant surveys to collect quantitative data about the intervention’s ability to improve dementia understanding.* | *Detailed descriptive and inferential statistics are provided for the surveys.* | *Control strategies are explained, and effect of prior dementia knowledge is discussed.* | *The intervention was delivered as planned.* | *Research aim is clearly stated at the end of the introduction.* | *Collected data is relevant to answer the question about the intervention’s enjoyability and impact on dementia attitudes.* |
| Liao (2022) | Y | Y | Y | Y | Y | Y | Y |
| *Justification* | *A sample of children were selected from a range of schools and 160/200 (80%) initially included children provided full data, with data about their varied sample characteristics shared.* | *Use of relevant surveys to collect quantitative data about the intervention’s ability to improve dementia understanding.* | *Pre- and post- intervention scores are provided for each survey question and 80% of the initial sample provided final outcome data.* | *Various control variables were factored into analyses and a control group was included.* | *Interventions were delivered as planned.* | *Research aims are clearly stated in the background section.* | *Collected data is relevant to answer the question about the intervention’s effect on dementia understanding.* |
| Masuda (2019) | U | Y | Y | U | Y | Y | Y |
| *Justification* | *No details of sample demographics* | *Dementia quizzes and questionnaires were appropriate tools for measuring the intervention’s impact.* | *All participants contributed to outcome measures* | *No detail of any control variables and volunteer bias.* | *Intervention was delivered as planned.* | *Research aims were communicated at the end of the introduction as assessing intervention efficacy.* | *Collected data is relevant to answer the question about assessing the intervention’s efficacy.* |
| **First Author (Year)** | **C1. Representativeness of Sample** | **C2. Outcome and Intervention Measurement Appropriateness** | **C3. Outcome Data Completeness** | **C4. Confounding Variable Control in Design and Analysis** | **C5. Intervention Administered as Planned** | **C6. Research Question Clarity** | **C7.**  **Collected Data’s Relevance for Question** |
| Noble (2015) | N | Y | Y | N | Y | Y | Y |
| *Justification* | *Convenience sample from mainly one cultural group.* | *Bespoke dementia knowledge tests were used to collect quantitative data about the intervention’s ability to teach children about Alzheimer’s disease.* | *91%-100% question response rate.* | *School had had previous stroke education which may have confounded the results about brain education.* | *Interventions were delivered as planned.* | *Research hypothesis is stated at the end of the introduction.* | *Collected data is relevant to answer the question about the intervention’s ability to teach children about Alzheimer’s disease.* |
| Parveen (2015) | U | U | N | U | Y | U | U |
| *Justification* | *Sample demographics not disclosed.* | *Measurement tools used not disclosed.* | *Full outcome data have not been provided from the quantitative results.* | *Confounding variables have not been discussed.* | *The intervention was delivered as planned.* | *Question is not clearly stated – inferred as evaluation of the intervention.* | *Not possible to assess as question not clearly stated.* |
| Sakai (2014) | N | Y | Y | N | Y | Y | Y |
| *Justification* | *Selectivity bias – parents had to sign children up through a database, so likely to be more invested in supporting children with the intervention.* | *Appropriate dementia surveys were used to assess the impact of reading story books for dementia education.* | *Very minimal missing data and extensive results section.* | *Selectivity bias reported.* | *Interventions were delivered as planned.* | *Research aims are stated at the end of the introduction.* | *Collected data is relevant to answer the question about the intervention’s ability to teach children about understanding storybooks’ dementia education impact.* |
| **First Author (Year)** | **C1. Representativeness of Sample** | **C2. Outcome and Intervention Measurement Appropriateness** | **C3. Outcome Data Completeness** | **C4. Confounding Variable Control in Design and Analysis** | **C5. Intervention Administered as Planned** | **C6. Research Question Clarity** | **C7.**  **Collected Data’s Relevance for Question** |
| Smith (2020) | Y | Y | Y | N | Y | Y | Y |
| *Justification* | *Recruited from various schools and varied demographics.* | *Dementia quizzes and interviews were appropriate tools for measuring the intervention’s impact.* | *Most (93%) of participants had data collected from them.* | *Dementia teaching in excess of the intervention was not controlled.* | *Intervention was delivered as planned.* | *Evaluating success and sustainability of interventions to guide future programme development.* | *Collected data is relevant to answer the question about assessing the intervention’s success and sustainability.* |

Results of Appraisals with the Mixed Methods Appraisal Tool (MMAT) for Quantitative Descriptive Studies (Hong et al., 2018)

| **First Author (Year)** | **Decisions and Justifications for Each Criterion of the MMAT for Quantitative Descriptive Studies** | | | | | | |
| --- | --- | --- | --- | --- | --- | --- | --- |
|  | **C1. Relevancy of sampling strategy for question** | **C2. Representativeness of sample** | **C3. Measurement appropriateness** | **C4. Lack of Nonresponse bias risk** | **C5. Statistical analysis appropriateness** | **C6. Research question clarity** | **C7.**  **Collected data’s relevance for question** |
| Nazir (2015) | Y | U | U | U | Y | U | N |
| *Justification* | *School sampling strategy was relevant for the study question.* | *Sample demographics not discussed.* | *Feedback form was an appropriate measure but not explained in sufficient detail.* | *Seemingly 25% of students did not fill in the feedback form but this was not discussed.* | *Descriptive statistics only used, which is appropriate based on sample size and data collected.* | *Question inferred as gauging effectiveness of awareness raising.* | *No quantitative data was shared about awareness improving as expected for the question.* |

Results of Appraisals with the Mixed Methods Appraisal Tool (MMAT) for Mixed Methods Studies (Hong et al., 2018)

| **First Author (Year)** | **Decisions and Justifications for Each Criterion of the MMAT for Mixed Methods Studies** | | | | | | |
| --- | --- | --- | --- | --- | --- | --- | --- |
|  | **C1. Mixed Methods Rationale** | **C2. Integration of Results** | **C3. Interpretation of Integration** | **C4. Qualitative and Quantitative Result Inconsistencies Addressed** | **C5. Appraisal of Individual Components** | **C6. Research Question Clarity** | **C7. Collected Data’s Relevance for Question** |
| Baker (2019) | Y | Y | Y | Y |  | Y | Y |
| *Justification* | *Reasons for quantitative and qualitative measures explained in methods.* | *How qualitative reports support quantitative results is discussed in the conclusion.* | *Discussion explains how the findings together suggest the programme is effective.* | *Results mainly complemented each other, with prior familiarity with dementia’s effect being discussed as a potential explanation for any inconsistencies across individuals.* | *See individual appraisal tools.* | *Research question is stated clearly in the introduction.* | *Both collected qualitative and quantitative data are relevant for the question.* |
| Burns (2020) | Y | Y | U | U |  | Y | Y |
| *Justification* | *Reasons for quantitative and qualitative measures explained in methods.* | *Discussion explains both the qualitative and quantitative results in relation to the research question.* | *It is not made clear how exactly the quantitative and qualitative results work together to answer the question.* | *Qualitative artwork results are not matched to survey scores so any differences between the two results cannot be deciphered.* | *See individual appraisal tools.* | *Research question is stated clearly in the introduction.* | *Both collected qualitative and quantitative data are relevant for the question.* |
| Chow (2018) | U | U | U | U |  | Y | Y |
| *Justification* | *Reasons for using quantitative and qualitative measures are not clearly explained.* | *Discussion does not clearly analyse both the qualitative and quantitative results.* | *It is not made clear how exactly the quantitative and qualitative results work together to answer the question.* | *It is not made clear how compatible the quantitative and qualitative results are.* | *See individual appraisal tools.* | *Research question is stated clearly in the introduction.* | *Both collected qualitative and quantitative data are relevant for the question.* |
| **First Author (Year)** | **C1. Mixed Methods Rationale** | **C2. Integration of Results** | **C3. Interpretation of Integration** | **C4. Qualitative and Quantitative Result Inconsistencies Addressed** | **C5. Appraisal of Individual Components** | **C6. Research Question Clarity** | **C7. Collected Data’s Relevance for Question** |
| Di Bona (2019) | Y | Y | Y | U |  | Y | Y |
| *Justification* | *Mixed-method approach is justified in the method section.* | *Discussion analyses both qualitative and quantitative data together under different subheadings.* | *Conclusion reflects on how scores increased and on how wider impacts were also discovered, interpreting the wider meaning of the results together.* | *It is not made clear how compatible the quantitative and qualitative results are.* | *See individual appraisal tools.* | *Research question is stated clearly in the introduction.* | *Both collected qualitative and quantitative data are relevant for the question.* |
| Masuda (2019) | U | U | U | U |  | Y | Y |
| *Justification* | *Not explained.* | *Results treated separately and not explicitly explained or integrated.* | *No clear interpretation of what both sets of results mean together.* | *Inconsistencies are not discussed explicitly.* | *See individual appraisal tools.* | *Question clearly stated as assessing intervention efficacy.* | *Data are relevant for the question of assessing intervention efficacy.* |
| Nazir (2015) | N | N | N | N |  | U | N |
| *Justification* | *Not explained.* | *Results treated separately and not explicitly explained or integrated.* | *No clear interpretation of what both sets of results mean together.* | *Inconsistencies are not discussed.* | *See individual appraisal tools.* | *Question only inferred as about the effectiveness of awareness raising.* | *Data about how effectively dementia awareness raising had occurred was not clearly discussed.* |
| Parveen (2015) | Y | U | U | U |  | U | U |
| *Justification* | *Explained as due to them evaluating the Dementia Detectives session.* | *Results are treated separately and not explicitly explained or integrated.* | *No clear interpretation of what both sets of results mean together.* | *Inconsistencies are not discussed.* | *See individual appraisal tools.* | *Question not clearly stated.* | *Not possible to assess as question not clearly stated.* |
| Smith (2020) | Y | Y | Y | Y |  | Y | Y |
| *Justification* | *Explained as due to provision of further insight than questionnaire alone.* | *Discussed together in the results and discussion.* | *Discuss how findings together may inform future developments or explain inconsistencies.* | *Discuss how quantitative results may differ due to the questionnaire not having scope to assess wider secondary effects of the intervention.* | *See individual appraisal tools.* | *Question clearly stated as assessing intervention success and sustainability.* | *Data are relevant for the question of assessing intervention success and sustainability.* |

Results of Appraisals with the Joanna Briggs Institute (JBI) Critical Appraisal Checklist for Qualitative Research (JBI, 2017)

| **First Author (Year)** | **Decisions and Justifications for Each Criterion of the JBI Critical Appraisal Checklist for Qualitative Research** | | | | | | | | | |
| --- | --- | --- | --- | --- | --- | --- | --- | --- | --- | --- |
|  | **C1. Congruity in Philosophical Perspective** | **C2. Congruity in Research Objective** | **C3. Congruity in Methods Used to Collect Data** | **C4. Congruity in Data Analysis** | **C5. Congruity in Interpretation of the Results** | **C6. Researcher Cultural or Theoretical Orientation** | **C7. Influence of the Researcher on the Research and Vice Versa Addressed** | **C8. Representativeness of the Participants’ Voices** | **C9. Ethical Approval** | **C10. Conclusions Drawn from the Analysis** |
| Baker (2019) | U | Y | Y | U | Y | Y | U | Y | Y | Y |
| *Justification* | *Not explicitly stated.* | *Focus group methodology related to study aims.* | *Data was collected as expected for a qualitative, focus group-based methodology.* | *Only positive responses were analysed; it is unclear whether negative responses were omitted or just not collected. Also, difficult to judge as methodological position/ philosophical perspective not clearly stated.* | *Data are used to understand the efficacy of the programme as planned and appropriate in relation to the methodology.* | *Backgrounds of all the authors are shared at the end of the paper.* | *The researchers do not clearly explain how their interpretation or leading of focus groups may have influenced the results.* | *Participant quotes are shared clearly.* | *Clear ethics statement.* | *Conclusions about programme efficacy are drawn based on the results.* |
| **First Author (Year)** | **C1. Congruity in Philosophical Perspective** | **C2. Congruity in Research Objective** | **C3. Congruity in Methods Used to Collect Data** | **C4. Congruity in Data Analysis** | **C5. Congruity in Interpretation of the Results** | **C6. Researcher Cultural or Theoretical Orientation** | **C7. Influence of the Researcher on the Research and Vice Versa Addressed** | **C8. Representativeness of the Participants’ Voices** | **C9. Ethical Approval** | **C10. Conclusions Drawn from the Analysis** |
| Burns (2020) | Y | Y | Y | Y | Y | U | U | Y | Y | Y |
| *Justification* | *Theoretical basis behind use of art as a qualitative measure is shared.* | *Changes in artwork pre- and post-intervention could be used to analyse the research objective of exploring how the intervention affects dementia understanding.* | *Data was collected as expected for an art-based qualitative study.* | *Data are analysed as expected for a study using an art-based theoretical basis.* | *Data are used to understand the efficacy of the programme as planned and appropriate in relation to the methodology.* | *Backgrounds of the authors are not shared in sufficient detail.* | *The authors do not clearly acknowledge or share mitigations for how their interpretations of the artworks could have influenced the results.* | *Artwork examples from participants are shared and described.* | *Ethics statement is provided.* | *Conclusions about changes in understanding are drawn based on the artworks.* |
| Chow (2018) | U | U | U | U | U | U | U | Y | Y | Y |
| *Justification* | *Not explicitly stated.* | *It is unclear how the qualitative data were collected.* | *It is unclear how the qualitative data were collected.* | *Difficult to judge as it is unclear how the qualitative data were collected, and methodological position/ philosophical perspective not clearly stated.* | *Discussion doesn’t clearly interpret the qualitative results.* | *Backgrounds of the authors are not shared in sufficient detail.* | *The authors do not clearly share how their involvement in the research may have influenced the results.* | *Quotes from participants are shared.* | *Ethics statement is provided.* | *Conclusions about programme success are drawn based on the analysis.* |
| **First Author (Year)** | **C1. Congruity in Philosophical Perspective** | **C2. Congruity in Research Objective** | **C3. Congruity in Methods Used to Collect Data** | **C4. Congruity in Data Analysis** | **C5. Congruity in Interpretation of the Results** | **C6. Researcher Cultural or Theoretical Orientation** | **C7. Influence of the Researcher on the Research and Vice Versa Addressed** | **C8. Representativeness of the Participants’ Voices** | **C9. Ethical Approval** | **C10. Conclusions Drawn from the Analysis** |
| Di Bona (2019) | U | Y | Y | U | Y | Y | U | Y | Y | Y |
| *Justification* | *Not explicitly stated.* | *Methodology related to aims to evaluate the program’s impact on children.* | *Data were collected as expected for a qualitative, interview- and focus group-based methodology.* | *Difficult to judge as methodological position/ philosophical perspective not clearly stated.* | *Conclusions are drawn in relation to the impact of the programme on student responses as planned and appropriate in relation to the methodology.* | *Author backgrounds are provided at the end of the paper.* | *The researchers do not clearly explain how their interpretation or leading of interviews and focus groups may have influenced the results.* | *Quotes and examples shared from participants.* | *Ethics statement provided.* | *Conclusions about programme impact are drawn based on the analysis.* |
| Farina (2020a) | U | Y | Y | U | Y | Y | Y | Y | Y | Y |
| *Justification* | *Not explicitly stated.* | *Focus group methodology related to study aims.* | *Data were collected as expected for a qualitative, focus group-based methodology.* | *Difficult to judge as methodological position/ philosophical perspective not clearly stated.* | *Data are used to understand the impact and perceptions of the programme as planned and appropriate in relation to the methodology.* | *Backgrounds of the authors are shared at the start of the paper and within the methods section.* | *A methods section dedicates itself to explaining how rigour was upheld in relation to researcher influences on results.* | *Quotes from participants are shared.* | *Ethics statement is provided.* | *Conclusions about programme impact are drawn based on the analysis.* |
| **First Author (Year)** | **C1. Congruity in Philosophical Perspective** | **C2. Congruity in Research Objective** | **C3. Congruity in Methods Used to Collect Data** | **C4. Congruity in Data Analysis** | **C5. Congruity in Interpretation of the Results** | **C6. Researcher Cultural or Theoretical Orientation** | **C7. Influence of the Researcher on the Research and Vice Versa Addressed** | **C8. Representativeness of the Participants’ Voices** | **C9. Ethical Approval** | **C10. Conclusions Drawn from the Analysis** |
| Gibson (2018) | Y | Y | Y | Y | N | Y | U | Y | Y | Y |
| *Justification* | *Person-centred research practice approach principles followed.* | *Methodology related to aims to evaluate the program.* | *Data were collected in a participant-personalised fashion as expected for a person-centred research methodology.* | *Data were analysed with many individual examples being drawn upon as expected for a person-centred research methodology.* | *Results are interpreted in a generalised fashion rather than in a person-centred approach.* | *Backgrounds of the authors are shared at the start of the paper.* | *The researchers do not clearly explain how their interpretation or leading of interviews may have influenced the results.* | *Quotes and examples shared from participants.* | *Ethics statement provided.* | *Conclusions about programme impact are drawn based on the analysis.* |
| Mastel-Smith (2022) | U | Y | Y | U | Y | Y | U | Y | U | Y |
| *Justification* | *Not explicitly stated.* | *Semi-structured interview method related to study aims.* | *Data was collected as expected for a qualitative, interview-based methodology.* | *Difficult to judge as methodological position/ philosophical perspective not clearly stated.* | *Conclusions are drawn in relation to programme feasibility and student responses to the programme as planned and appropriate in relation to the methodology.* | *Backgrounds of the authors are shared at the start of the paper.* | *The authors do not clearly share how their interpretation or leading of the interviews may have influenced the results.* | *Participant quotes are shared throughout.* | *An ethics statement is not clearly shared.* | *Conclusions about feasibility and student responses are drawn based on the analysis.* |
| **First Author (Year)** | **C1. Congruity in Philosophical Perspective** | **C2. Congruity in Research Objective** | **C3. Congruity in Methods Used to Collect Data** | **C4. Congruity in Data Analysis** | **C5. Congruity in Interpretation of the Results** | **C6. Researcher Cultural or Theoretical Orientation** | **C7. Influence of the Researcher on the Research and Vice Versa Addressed** | **C8. Representativeness of the Participants’ Voices** | **C9. Ethical Approval** | **C10. Conclusions Drawn from the Analysis** |
| Masuda (2019) | U | U | U | U | U | U | U | U | Y | Y |
| *Justification* | *Not disclosed explicitly.* | *Qualitative methodology too unclear to assess.* | *Qualitative methodology too unclear to assess.* | *Qualitative methodology too unclear to assess.* | *Qualitative methodology too unclear to assess.* | *Insufficient author details provided.* | *Not discussed explicitly.* | *Very limited participant quotes shared.* | *Ethics statement shared.* | *Conclusions relate to results.* |
| Nazir (2015) | U | U | U | U | U | Y | U | Y | N | N |
| *Justification* | *Not disclosed explicitly.* | *Method did not measure the inferred question of awareness raising.* | *Qualitative methodology too unclear to assess.* | *Qualitative methodology too unclear to assess, for example, nature of feedback form questions not disclosed.* | *Qualitative methodology too unclear to assess.* | *Author details are provided at the end of the paper.* | *Not discussed explicitly.* | *Participant quotes shared.* | *Ethics statement not shared.* | *Concluded future steps do not explicitly arise from the results.* |
| Parveen (2015) | U | U | U | U | U | Y | U | N | N | Y |
| *Justification* | *Not disclosed.* | *Question unclear.* | *Qualitative methodology too unclear to assess.* | *Difficult to judge as methodological position/ philosophical perspective not clearly stated.* | *Qualitative methodology too unclear to assess.* | *Author backgrounds are provided at the end of the paper.* | *Not discussed explicitly.* | *Participant quotes not shared.* | *Ethics statement not shared.* | *Conclusions relate to results.* |
| **First Author (Year)** | **C1. Congruity in Philosophical Perspective** | **C2. Congruity in Research Objective** | **C3. Congruity in Methods Used to Collect Data** | **C4. Congruity in Data Analysis** | **C5. Congruity in Interpretation of the Results** | **C6. Researcher Cultural or Theoretical Orientation** | **C7. Influence of the Researcher on the Research and Vice Versa Addressed** | **C8. Representativeness of the Participants’ Voices** | **C9. Ethical Approval** | **C10. Conclusions Drawn from the Analysis** |
| Pawlicka  (2023) | Y | Y | N | N | Y | U | U | Y | Y | Y |
| *Justification* | *Constructivist grounded theory adhered to.* | *Methodology justified for research objective.* | *Data not collected iteratively.* | *Data not analysed iteratively.* | *Results interpreted as appropriate for methodology.* | *Author background detail insufficient.* | *Not discussed explicitly.* | *Quotes from participants justifying themes are shared.* | *Ethics statement provided.* | *Conclusions relate to the results.* |
| Ritchie (2023) | U | Y | Y | U | Y | U | U | Y | Y | Y |
| *Justification* | *Alluded to when discussing vignettes. Not explicitly mentioned in relation to analysis.* | *Qualitative methodology using video methods and focus groups relevant for aims.* | *Data were collected as expected for a qualitative methodology using video methods and focus groups.* | *Difficult to judge as methodological position/ philosophical perspective not clearly stated.* | *Best practice conclusions are drawn as planned and appropriate in relation to the methodology.* | *Author backgrounds are not provided in sufficient detail.* | *Risk of researcher influence during focus groups is not explained.* | *Quotes are shared from participants and category headings reflected participant statements.* | *Ethics statement provided.* | *Conclusions about best teaching practices are drawn based on the analysis.* |
| Smith (2020) | U | Y | Y | U | Y | U | U | U | Y | Y |
| *Justification* | *Not discussed explicitly.* | *Semi-structured interview method related to study aims.* | *Data was collected as expected for a qualitative, interview-based methodology.* | *Difficult to judge as methodological position/ philosophical perspective not clearly stated.* | *Data are used as planned and appropriate in relation to the methodology.* | *Insufficient author details provided.* | *Not discussed explicitly.* | *Not enough quotes shared to warrant a clear participant voice.* | *Ethics statement shared.* | *Conclusions relate to the results.* |

Results of Level of Evidence Assessments with ConQual (Munn et al., 2014).

**Dependability**

*See the above results for appraisals with the JBI critical appraisal checklist for justifications of the decisions made for the five criteria below.*

| **First Author (Year)** | **C1. Congruity in Research Objective** | **C2. Congruity in Methods Used to Collect Data** | **C3. Method and Data Analysis Congruity** | **C4. Researcher Beliefs Shared** | **C5. Researcher Influence Addressed** | **Score** | **Ranking** |
| --- | --- | --- | --- | --- | --- | --- | --- |
| Baker (2019) | Y | Y | U | Y | U | 3Y | ↓ |
| Burns (2020) | Y | Y | Y | U | U | 3Y | ↓ |
| Chow (2018) | U | U | U | U | U | 0Y | ↓↓ |
| Di Bona (2019) | Y | Y | U | Y | U | 3Y | ↓ |
| Farina (2020a) | Y | Y | U | Y | Y | 4Y | ↔ |
| Gibson (2018) | Y | Y | Y | Y | U | 4Y | ↔ |
| Mastel-Smith (2022) | Y | Y | U | Y | U | 3Y | ↓ |
| Masuda (2019) | U | U | U | U | U | 0Y | ↓↓ |
| Nazir (2015) | U | U | U | Y | U | 1Y | ↓↓ |
| Parveen (2015) | U | U | U | Y | U | 1Y | ↓↓ |
| Pawlicka (2023) | Y | N | N | N | N | 1Y | ↓↓ |
| Ritchie (2023) | Y | Y | U | U | U | 2Y | ↓ |
| Smith (2020) | Y | Y | U | U | U | 2Y | ↓ |

**Credibility**

| **First Author (Year)** | **Ranking** | **Justification** |
| --- | --- | --- |
| Baker (2019) | Mixture of unequivocal and equivocal | Most findings are supported by quotes except from the finding that children enjoyed the programme in general. |
| Burns (2020) | Equivocal | All findings could be debated as they rest on subjective interpretations of artworks. |
| Chow (2018) | Equivocal | Limited quotational detail is provided about the qualitative findings but they are plausible. |
| Di Bona (2019) | Mixture of unequivocal and equivocal | Some findings are supported with justifying quotes from children whilst others are more equivocal and, for example, rely on research interpretations of children’s expressions. |
| Farina (2020a) | Mixture of unequivocal and equivocal | Most findings are supported by quotes except there is not a clear link between the quote and the interactivity finding. |
| Gibson (2018) | Mixture of unequivocal and equivocal | Most findings could be debated as they rest on subjective interpretations of artworks and synonym naming as opposed to explicit explanations about the works from the creators. Some findings are unequivocal due to the provided support. |
| Mastel-Smith (2022) | Mixture of unequivocal and equivocal | Most findings are supported by quotes but there isn’t an explicit justification of why the programme was deemed feasible. |
| Masuda (2019) | Plausible/Unsupported | Findings are plausible but unsupported as there is limited detail as to how they were collected. |
| Nazir (2015) | Plausible/Unsupported | Findings are plausible but unsupported as there is limited detail as to how they were collected |
| Parveen (2015) | Plausible/Unsupported | Findings are plausible but unsupported. There is low detail as to how they were collected and limited supporting quotes. |
| Pawlicka (2023) | Mixture of unequivocal and equivocal | Most findings are supported by quotes except from the finding that children could use art to express dementia learning. |
| Ritchie (2023) | Mixture of unequivocal and equivocal | Mixture of unequivocal and equivocal findings. |
| Smith (2020) | Equivocal | Not enough participant voice shared to confidently support all statements made. |

**ConQual Overall Results**

| **First Author (Year)** | **Type of Research** | **Pre-ranking** | **Dependability** | **Credibility** | **ConQual Level of Evidence** | **Comments** |
| --- | --- | --- | --- | --- | --- | --- |
| Baker (2019) | Mixed Methods | High | ↓ | ↓ | Low | Dependability downgraded one level as only 2-3 criteria were positive. Credibility downgraded one level as there was a mixture of unequivocal and equivocal findings. |
| Burns (2020) | Mixed Methods | High | ↓ | ↓↓ | Very Low | Dependability downgraded one level as only 2-3 criteria were positive. Credibility downgraded two levels as the art-based data demonstrating dementia understanding changing through art was found to be equivocal. |
| Chow (2018) | Mixed Methods | High | ↓↓ | ↓↓ | Very Low | Dependability downgraded two levels as 0 criteria were supported. Credibility downgraded two levels as all the findings were equivocal. |
| Di Bona (2019) | Qualitative | High | ↓ | ↓ | Low | Dependability downgraded one level as only 2-3 criteria were positive. Credibility downgraded one level as there was a mixture of unequivocal and equivocal findings. |
| Farina (2020a) | Qualitative | High | ↔ | ↓ | Moderate | Dependability unchanged since 4-5 criteria were positive. Credibility downgraded one level as there was a mixture of unequivocal and equivocal findings. |
| Gibson (2018) | Qualitative | High | ↔ | ↓ | Moderate | Dependability unchanged since 4-5 criteria were positive. Credibility downgraded one level as there was a mixture of unequivocal and equivocal findings. |
| Mastel-Smith (2022) | Qualitative | High | ↓ | ↓ | Low | Dependability downgraded one level as only 2-3 criteria were positive. Credibility downgraded one level as there was a mixture of unequivocal and equivocal findings. |
| Masuda (2019) | Qualitative | High | ↓↓ | ↓↓↓ | Very Low | Dependability downgraded two levels as only 1 criteria was supported. Credibility downgraded three levels as results were a mixture of equivocal and unsupported. |
| Nazir (2015) | Qualitative | High | ↓↓ | ↓↓↓ | Very Low | Dependability downgraded two levels as only 1 criteria was supported. Credibility downgraded three levels as results were a mixture of equivocal and unsupported. |
| Parveen (2015) | Qualitative | High | ↓↓ | ↓↓↓ | Very Low | Dependability downgraded two levels as only 1 criteria was supported. Credibility downgraded three levels as results were a mixture of equivocal and unsupported. |
| Pawlicka (2023) | Qualitative | High | ↓↓ | ↓ | Very Low | Dependability downgraded one level as only 2-3 criteria were positive. Credibility downgraded one level as there was a mixture of unequivocal and equivocal findings. |
| Ritchie (2023) | Qualitative | High | ↓ | ↓ | Low | Dependability downgraded one level as only 2-3 criteria were positive. Credibility downgraded one level as there was a mixture of unequivocal and equivocal findings. |
| Smith (2020) | Qualitative | High | ↓ | ↓↓ | Very Low | Dependability downgraded one level as only 2-3 criteria were positive. Credibility downgraded two levels as qualitative findings were found to be equivocal. |

Results of Level of Evidence Assessments with the Grading of Recommendations Assessment, Development and Evaluation (GRADE) system (Ryan and Hill, 2016).

| **First Author (Year)** | **Decisions and Comments** | **Pre-ranking** | **Risk of bias** | **Inconsistency** | **Indirectness** | **Imprecision** | **Publication Bias** | **Large magnitude of effect** | **Dose response** | **Effect of all plausible confounding variables** | **GRADE Level of Evidence** |
| --- | --- | --- | --- | --- | --- | --- | --- | --- | --- | --- | --- |
| Baker (2019) | *Ranking* | Low | ↔ | ↔ | ↔ | ↓ | ↔ | ↔ | ↔ | ↑ | Low |
|  | *Comments* | Not an RCT. | Plausible bias due to lack of blinding but unlikely to seriously alter the results. | Inconsistent results explained and controlled for by familiarity factor so no issues. | Population, intervention, comparison group and outcome measures were all applicable to real life so no issues. | Sample size too small to calculate precise effect sizes and no confidence intervals. | Undetected. | Undetected. | Undetected. | Effect still found when confounding variables (familiarity) are controlled for. |  |
| Burns (2020) | *Ranking* | Low | ↓ | ↔ | ↔ | ↓ | ↔ | ↔ | ↔ | ↔ | Very Low |
|  | *Comments* | Not an RCT. | Pre- and post-data collected depended on classroom teacher’s enthusiasm. | Inconsistent results explained by ceiling effect. | Population, intervention and outcome measures were all applicable to real life so no issues. | Sample size too small to calculate precise effect sizes and no confidence intervals. | Undetected. | Undetected. | Undetected. | Undetected. |  |
| **First Author (Year)** | **Decisions and Comments** | **Pre-ranking** | **Risk of bias** | **Inconsistency** | **Indirectness** | **Imprecision** | **Publication Bias** | **Large magnitude of effect** | **Dose response** | **Effect of all plausible confounding variables** | **GRADE Level of Evidence** |
| Chow (2018) | *Ranking* | Low | ↓ | ↓ | ↔ | ↓ | ↔ | ↔ | ↔ | ↔ | Very Low |
|  | *Comments* | Not an RCT. | Self-selected sample. | Quantitative scores not disclosed. | Population, intervention and outcome measures were all applicable to real life so no issues. | Sample size too small to calculate precise effect sizes and no confidence intervals. | Undetected. | Undetected. | Undetected. | Undetected. |  |
| Di Bona (2019) | *Ranking* | Low | ↓ | ↔ | ↔ | ↓ | ↔ | ↔ | ↔ | ↔ | Very Low |
|  | *Comments* | Not an RCT. | Representativeness lost as children were specially selected for the study. | Inconsistent results discussed as a product of questionnaire type. | Population, intervention, and outcome measures were all applicable to real life so no issues. | Sample size too small to calculate precise effect sizes and large confidence intervals. | Undetected. | Undetected. | Undetected. | Undetected. |  |
| Farina (2020b) | *Ranking* | Low | ↔ | ↔ | ↔ | ↓ | ↔ | ↔ | ↔ | ↑ | Low |
|  | *Comments* | Not an RCT. | Plausible bias due to lack of blinding but unlikely to seriously alter the results. | Inconsistent results explained by social desirability bias and halo effect. | Population, intervention, comparison group and outcome measures were all applicable to real life so no issues. | Sample size too small to calculate precise effect sizes and large confidence intervals. | Undetected. | Undetected. | Undetected. | Effect still not found when confounding variables are controlled for in the analysis. |  |
| **First Author (Year)** | **Decisions and Comments** | **Pre-ranking** | **Risk of bias** | **Inconsistency** | **Indirectness** | **Imprecision** | **Publication Bias** | **Large magnitude of effect** | **Dose response** | **Effect of all plausible confounding variables** | **GRADE Level of Evidence** |
| Liao (2022) | *Ranking* | Low | ↔ | ↔ | ↔ | ↓ | ↔ | ↔ | ↔ | ↑ | Low |
|  | *Comments* | Not an RCT. | Blinding used. | Inconsistent results discussed as a product of intervention length. | Population, intervention, comparison group and outcome measures all applied to real life. | Sample size too small to calculate precise effect sizes and large confidence intervals. | Undetected. | Undetected. | Undetected. | Effect still found when potential confounding variables are controlled for. |  |
| Masuda (2019) | *Ranking* | Low | ↓ | ↓ | ↓ | ↓ | ↔ | ↔ | ↔ | ↔ | Very Low |
|  | *Comments* | Not an RCT. | High as volunteer sampling. | Inconsistencies are not discussed or included. | Population composition not disclosed. | Sample size too small to calculate precise effect sizes. | Undetected. | Undetected. | Undetected. | Undetected. |  |
| Nazir (2015) | *Ranking* | Low | ↓ | ↓ | ↓ | ↓ | ↔ | ↔ | ↔ | ↔ | Very Low |
|  | *Comments* | Not an RCT. | High as school sampling strategy not explained. | Inconsistencies are not discussed or included. | Population composition not disclosed. | Sample size too small to calculate precise effect sizes. | Undetected. | Undetected. | Undetected. | Undetected. |  |
| Noble (2015) | *Ranking* | Low | ↓ | ↔ | ↓ | ↓ | ↔ | ↔ | ↔ | ↔ | Very Low |
|  | *Comments* | Not an RCT. | High due to confounding variables and convenience sampling method. | Inconsistent results explained by confounding variable of previous stroke education. | Population included children mostly from a minority ethnic group. | Sample size too small to calculate precise effect sizes. | Undetected. | Undetected. | Undetected. | Undetected. |  |
| **First Author (Year)** | **Decisions and Comments** | **Pre-ranking** | **Risk of bias** | **Inconsistency** | **Indirectness** | **Imprecision** | **Publication Bias** | **Large magnitude of effect** | **Dose response** | **Effect of all plausible confounding variables** | **GRADE Level of Evidence** |
| Parveen (2015) | *Ranking* | Low | ↓ | ↓ | ↓ | ↓ | ↔ | ↔ | ↔ | ↔ | Very Low |
|  | *Comments* | Not an RCT. | High due to undisclosed sampling methods or bias mitigation. | Inconsistencies are not discussed or included. | Population composition not disclosed. | Sample size too small to calculate precise effect sizes. | Undetected. | Undetected. | Undetected. | Undetected. |  |
| Sakai (2014) | *Ranking* | Low | ↓ | ↔ | ↓ | ↓ | ↔ | ↔ | ↔ | ↔ | Very Low |
|  | *Comments* | Not an RCT. | High due to selectivity bias. | Statistics for inconsistencies where overall trends were found are provided. | Selectivity bias limits population directness. | Sample size too small to calculate precise effect sizes. | Undetected. | Undetected. | Undetected. | Undetected. |  |
| Smith (2020) | *Ranking* | Low | ↔ | ↔ | ↔ | ↓ | ↔ | ↔ | ↔ | ↔ | Very Low |
|  | *Comments* | Not an RCT. | Plausible bias due to lack of blinding but unlikely to seriously alter results. | Inconsistencies are discussed as a result of prior familiarity. | Population, intervention, and outcome measures were all applicable to real life so no issues. | Sample size too small to calculate precise effect sizes. | Undetected. | Undetected. | Undetected. | Undetected. |  |

APPENDIX 6: Intervention Details

| **Alpha Code: First Author (Year)** | **Evaluated Intervention’s Name: Summary** | **Intervention Length** | **Intervention Structure Detail** | **Intervention Topic Focus** | **Intervention Aim** |
| --- | --- | --- | --- | --- | --- |
| A: Baker (2019) | Kids4Dementia: teacher-led multimedia DEP. | 150 minutes delivered over 4 to 10 weeks. | A website supporting seven dementia education modules is delivered to the children within class time by their teachers. Modules were linked by an animated narrative of siblings and their grandfather. Modules also included videos of adults living with dementia and their child relatives. Modules were supplemented with class or homework activities including role-plays or drawings. | Dementia definition. Interacting and helping people living with dementia. Nursing homes. Causes of dementia. Keeping brains healthy. Experience of living with dementia for patients and family. | To improve dementia literacy. |
| B and N: Burns (2020)  and  Pawlicka  (2023) | Project DARE: arts-based DEP. | Three sessions delivered one day a week, over a three-week period. Each session lasted one hour. | Lesson 1: An art lesson, delivered by local artists where children had to draw a memory. Lesson 2: Dementia education delivered by the classroom teacher. Resources used included a song, a web-based brain anatomy resource. Lesson 3: Art follow-up lesson delivered by local artists where children had to modify their drawing in line with how someone with dementia may experience it. | Prior understanding of dementia. Participant children’s personal experiences of dementia. Dementia definition. Brain changes in dementia. How to help people living with dementia. | To increase understanding about dementia. |
| C: Chow (2018) | Dementia Awareness Program: intergenerational program. | Weekly hour-long visits with a person living with dementia for a total of 15 weeks. | Each visit began with a half-hour teatime, followed by a half-hour of  brain-stimulating activities including puzzles, music therapy and games. | Getting to know a person with dementia through conversation and activities. | To increase knowledge and appreciation of dementia, reducing the stigma surrounding the illness, and provide  opportunity for students to volunteer with and enrich  the lives of people with dementia. |
| D: Di Bona (2019) | Adopt a Care Home: teacher-led DEP with intergenerational experience for some students. | Several care home visits of undisclosed length. One school term of regular dementia lessons. | Children are taught about dementia in school lessons, which included case studies, experiential games, and teaching of facts. As many children as practicable also participating in care home intergenerational sessions where they complete lifestory books with people living with dementia. | What dementia is. The effects of living with dementia. | To increase children’s dementia awareness and improve wellbeing and community participation for people living with dementia in care homes. |
| E and F: Farina (2020a; 2020b) | Dementia Friends: dementia information session. | 45-60 minute long single session. | Each session is run by a volunteer and covers five key messages through a mixture of question and answers, analogies and interactive tasks. No videos of or live people with dementia or carers are included. The sessions end with participants committing to a dementia friendly action and receiving a ‘Dementia Friend’ status and badge. | Dementia versus natural aging. Causes of dementia and the brain. Dementia symptoms beyond memory loss. Living well with dementia. The person beyond their dementia. | Dementia education and awareness initiative aimed at reducing stigma. |
| G: Gibson (2018) | Adapted Dementia Friends: dementia information session. | 150 minute long session with a break consisting of four 15 minute long interactive sessions. | Each interactive session was delivered in schools by student facilitators and consisted of short activities discussing dementia. | Dementia definition. Feelings and self-expression of people living with dementia. Coping when you know someone with dementia. | To raise awareness about dementia, inform how people can live well with dementia, and consider how society can make a positive difference to local people living with dementia. To adapt the Dementia Friends intervention to make it more interactive. |
| H: Liao (2022) | Intergenerational intervention with interaction via exergaming or activity companionship. | Weekly 40 minute long sessions for either 5 or 8 weeks depending on intervention group allocation. | Each intervention session involved a participant working alone or together in pairs with an older adult with dementia. The sessions had an introduction, activity or conclusion. The exergaming intervention activities exergames were video games requiring physical motion, visual perception and cooperation. The companion intervention activities were regular daily activities hosted in the daycare centre such as singing or painting. | Interacting with a person with dementia. | To improve attitudes and understanding towards people living with dementia. |
| I: Mastel-Smith (2022) | Online Dementia Bootcamp: virtual dementia education platform with online live sessions. | Live sessions were held once a week for six weeks and for 90 minutes per session. | Pre-work, online readings and videos were provided in an online learning management system. Weekly sessions generally followed the structure of a 45 minute presentation by the programme facilitators (three nurses and an occupational therapist) which included discussions and small group activities. This was then followed by a 45 minute session with a guest speaker, including people living with dementia or care partners. Students also were allocated a person living with dementia to talk to privately in a video call. | Introduction to dementia. Preventing dementia. Alzheimer’s disease. Behavioural and psychological symptoms of dementia. Lewy body dementia. Communicating with a person with dementia. Binswanger dementia. Supporting families living with dementia. | To improve dementia knowledge and perceptions of dementia. |
| J: Masuda (2019) | Pepper: Robot-mediated DEP. | A training course of undisclosed length. | Pepper the robot mediates the learning session by acting as both a tutor and an older person with dementia. Pepper teaches children about dementia and quizzes them on their understanding. | Dementia as a disease. Dementia’s impacts on memory, learning and thinking. | To improve dementia awareness and knowledge. |
| K: Nazir (2015) | Dementia Awareness-Raising and Education Project: age-adapted dementia teaching sessions. | Five hour-long sessions delivered over the course of a year. | Material was presented in age-appropriate teaching sessions which was delivered via teaching presentations, a brain model, and role plays. Sessions were delivered in the classroom by the researchers to groups of 10. | Dementia causes and symptoms. Emotional and physical problems experienced by people living with dementia and their carers. Care services. The brain and dementia. Diagnosis of dementia through standardised exams. | To teach about dementia and the needs of people living with it and their carers. |
| L: Noble (2015) | Old SCHOOL Hip-Hop: arts-based Alzheimer’s disease education programme with a mnemonic of key symptoms. | Sessions were an hour long and delivered over three consecutive days. | Sessions were delivered by the researchers in the classroom and included dance, culturally appropriate and age-appropriate music (rap about Alzheimer’s disease), role play performances, comic book pages, and short animated films. A mnemonic was developed as a memory aid for dementia symptoms: FLOW (forget, lose, overlook, write/wander). Activities in the session were centred around the mnemonic. For example, students could use it to diagnose different fictional adult characters with dementia or normal ageing. | Introduction to dementia. Alzheimer’s disease and the brain. Discussing dementia with parents. Normal versus pathologic ageing. Early symptom detection. | To improve dementia health literacy and to enable children to be able to teach about Alzheimer’s disease at home. |
| M: Parveen (2015) | Dementia Detectives: detective-themed dementia awareness session. | A one-hour session. | The session was delivered by the researcher in the classroom. It included a teaching presentation and several ‘operations’ which involved students exploring material related to dementia as ‘detectives’. Children are split up into teams to conduct the activities, and then group discussions are facilitated. | Dementia definition. Nature of dementia. Dementia risk factors. Symptom detection. Uniqueness of dementia experience. Dementia stigma. Case studies of people living with dementia. Supporting someone living with dementia. | To improve dementia awareness, advocacy and attitudes. |
| O: Ritchie (2023) | Class in a Bag: Dementia awareness session delivered using a resource bag. | A single, 80 minute session. | The session was delivered by the teacher and two researchers in the school. Three activities were delivered, and children could rotate around each activity. The first activity was learning about the brain through a brain model. The second activity was understanding the experience of dementia through a simulation where children had to find cards in a deck whilst wearing obscured lens goggles, gloves and listening to a white noise mp3 player. The last activity was exploring technology solutions and reminiscence aids for people living with dementia. | Understanding the brain. Understanding the experience of dementia. How to help someone with dementia. | To improve dementia awareness to support with children being socially responsible citizens. |
| P: Sakai (2014) | Allie Learns about Alzheimer’s Disease: Alzheimer’s disease storybook (Gosselin, 2001). | 1-1.5 hour session. | Parents read the storybook to their child, generally lasting 12-15 minutes. Following a short break where outcome measures were collected, parents discussed the book with their child for 12 minutes. Parents were given a discussion guide to facilitate this. Parents were notified by researchers if they missed a discussion question. | Cognitive and emotional symptoms of Alzheimer’s disease. Diagnosis and treatment of Alzheimer’s disease. Carer responses to people living with Alzheimer’s disease. | To improve knowledge, attitudes and willingness to interact with people living with dementia. |
| Q: Smith (2020) | Adapted Kids4Dementia: teacher-led multimedia DEP with or without intergenerational experience. | One 45 minute long weekly lesson was delivered each week for 8 weeks. 45 minute long excursions began for the eligible groups from week 3 of the program. | The programme was modified from Kids4Dementia by adding new age-appropriate activities. Content was delivered through videos, activity booklets, brain models, question and answer sessions with a geriatrician, and brainstorming. The intergenerational excursions were based around a shopping theme where experiences of shopping were discussed with the older adults. Activities during the excursions were conducted in groups of the older adults and the children and led by Enabling Confidence at Home (ECH) activity and lifestyle staff, and commissioned community artists. Examples included flowerpot planting, 8-ball, dyeing fabric (cyanotype) to make re-usable shopping bags and singing. Children who did not go on the excursions completed the same activities at school without the older adults. | Dementia definition. Experience of having dementia. Nursing homes. Causes of dementia. Brain health. Technology aids for people with dementia. Environmental adaptations to support a person living with dementia. Family experiences of dementia. | To improve dementia knowledge and attitudes. |
